# Supplementary figures and images for: Risk of recurrence and bleeding in patients with cancer-associated venous thromboembolism in the direct oral anticoagulants era: Findings from the TULIPE registry
Source: PLoS One. 2025 Jul 29;20(7):e0329025. doi: 10.1371/journal.pone.0329025 (PMC12306744; doi:10.1371/journal.pone.0329025)

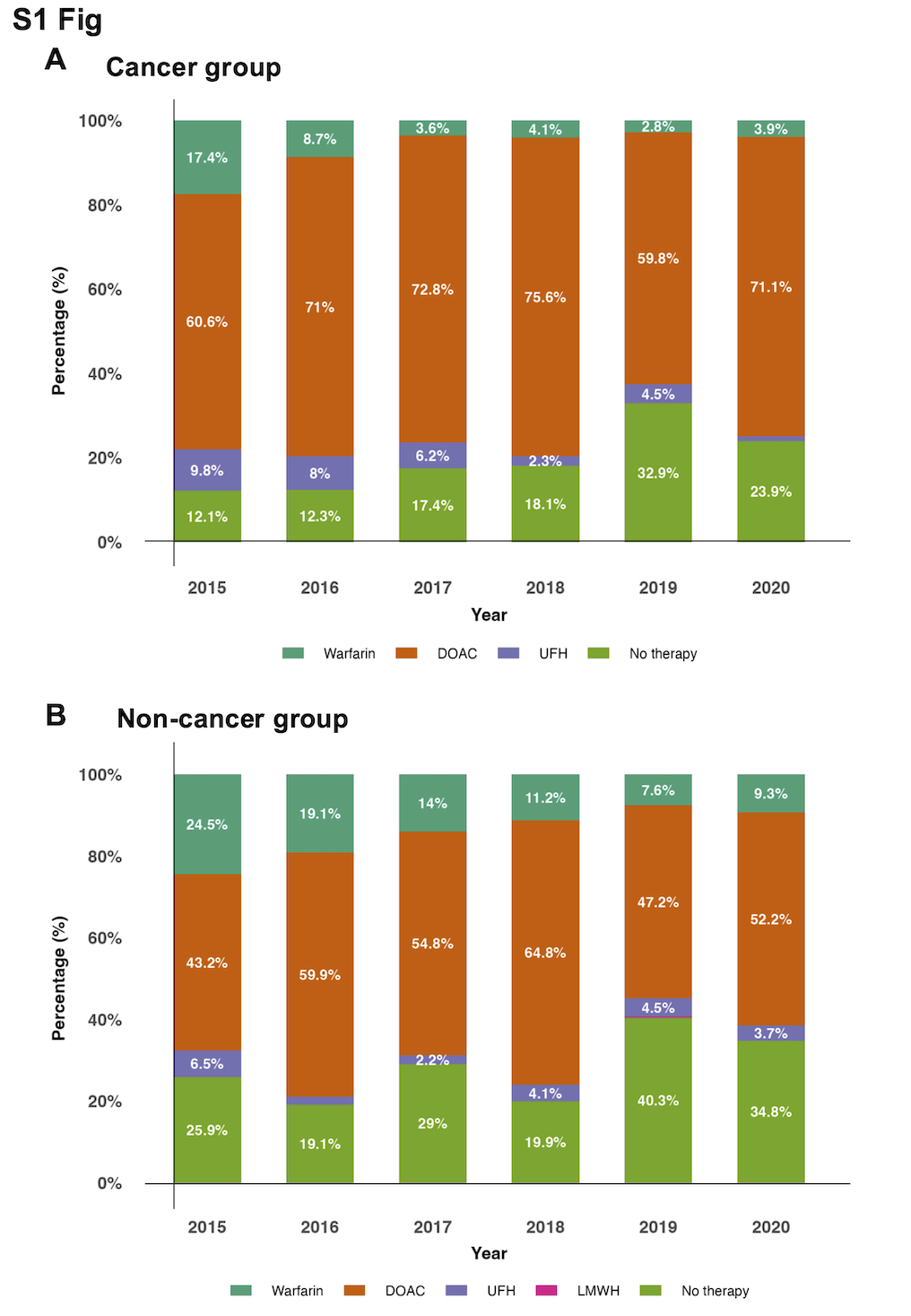

Supplement: S1 Fig — Cancer (A) and non-cancer (B) groups. DOAC, direct oral anticoagulant; LMWH, low-molecularweight heparin; UFH, unfractionated heparin. (TIF) [file pone.0329025.s001.tif]

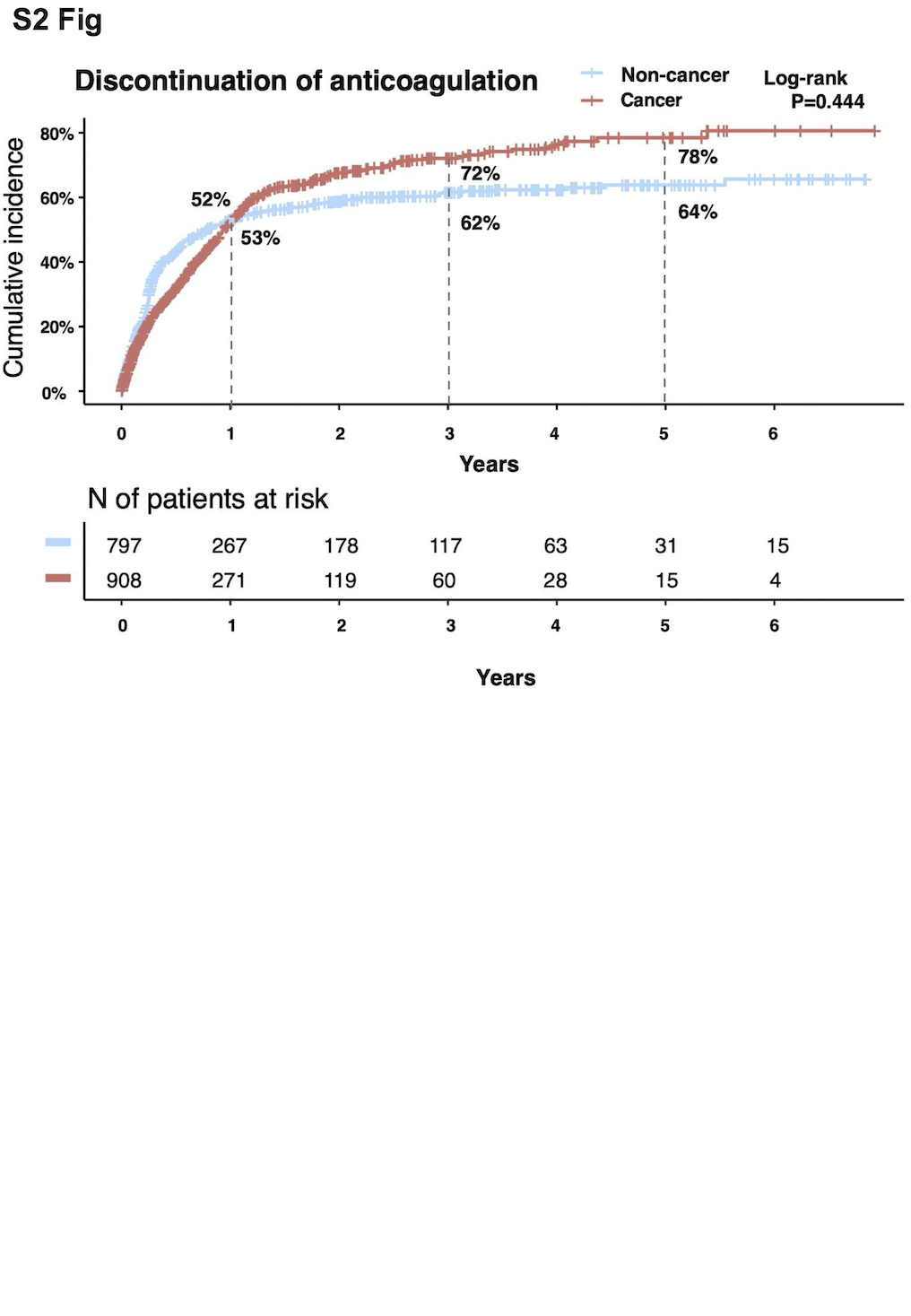

Supplement: S2 Fig — (TIF) [file pone.0329025.s002.tif]

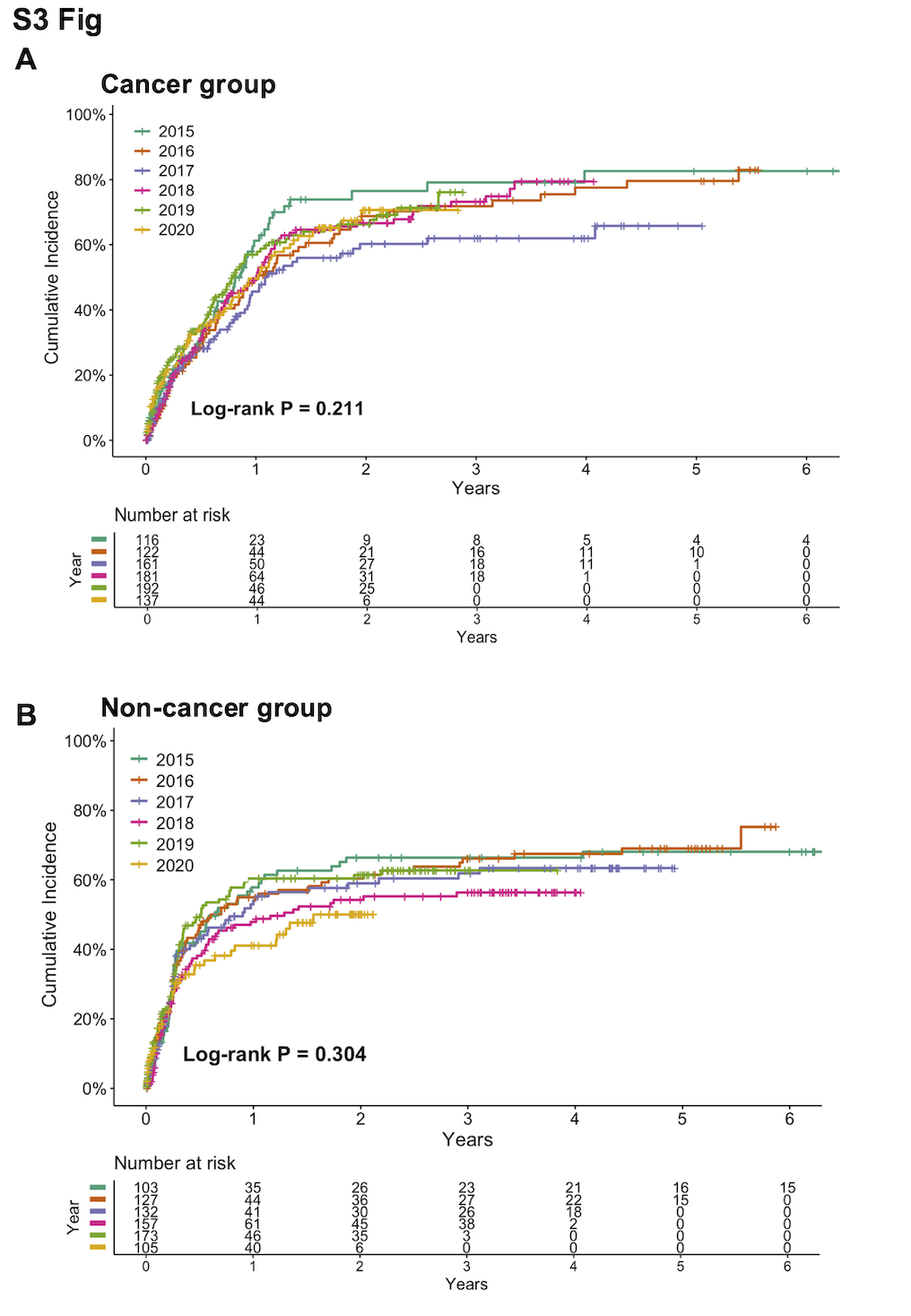

Supplement: S3 Fig — Cancer (A) and non-cancer (B) groups. (TIF) [file pone.0329025.s003.tif]

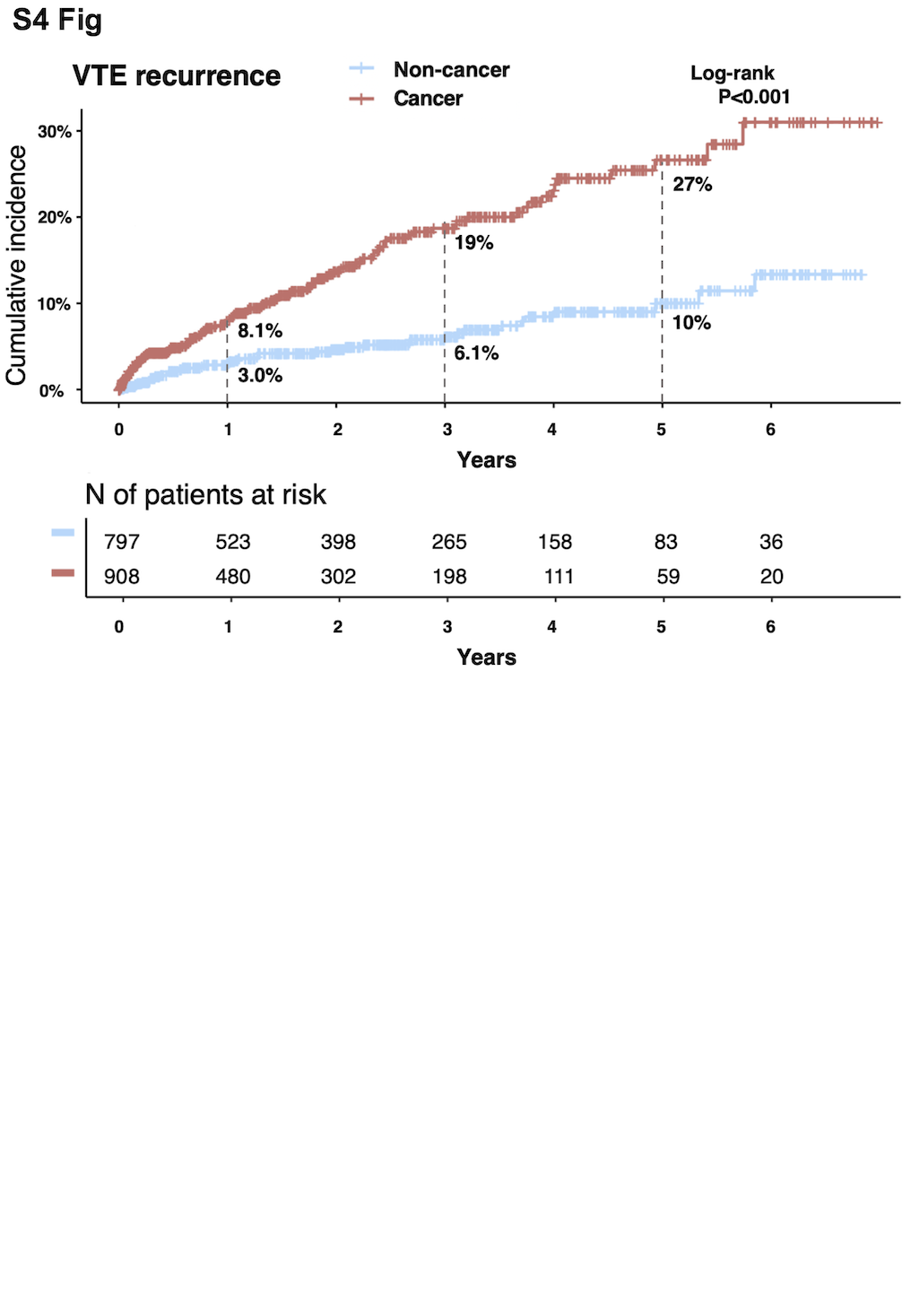

Supplement: S4 Fig — VTE, venous thromboembolism. (TIF) [file pone.0329025.s004.tif]

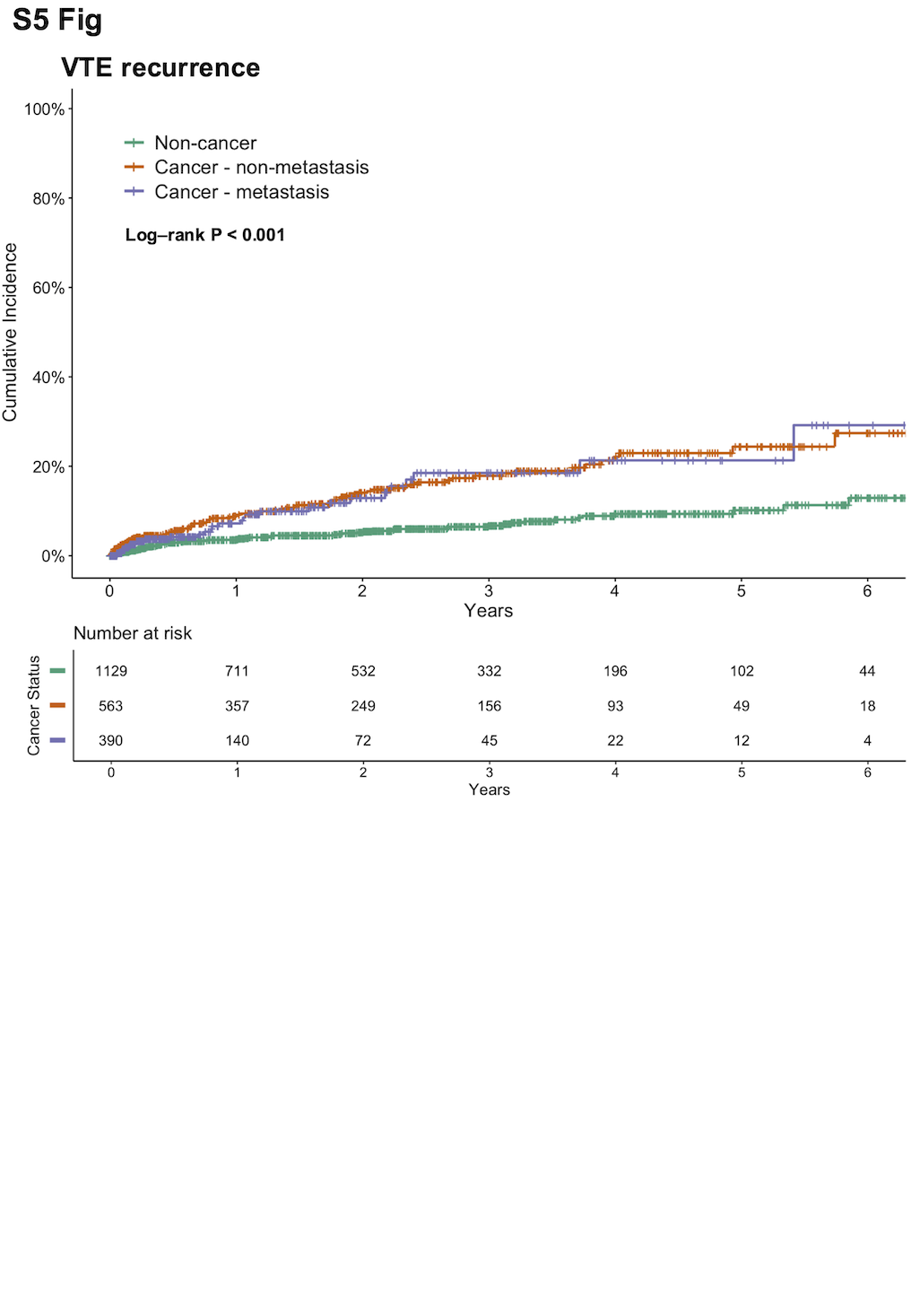

Supplement: S5 Fig — VTE, venous thromboembolism. (TIF) [file pone.0329025.s005.tif]

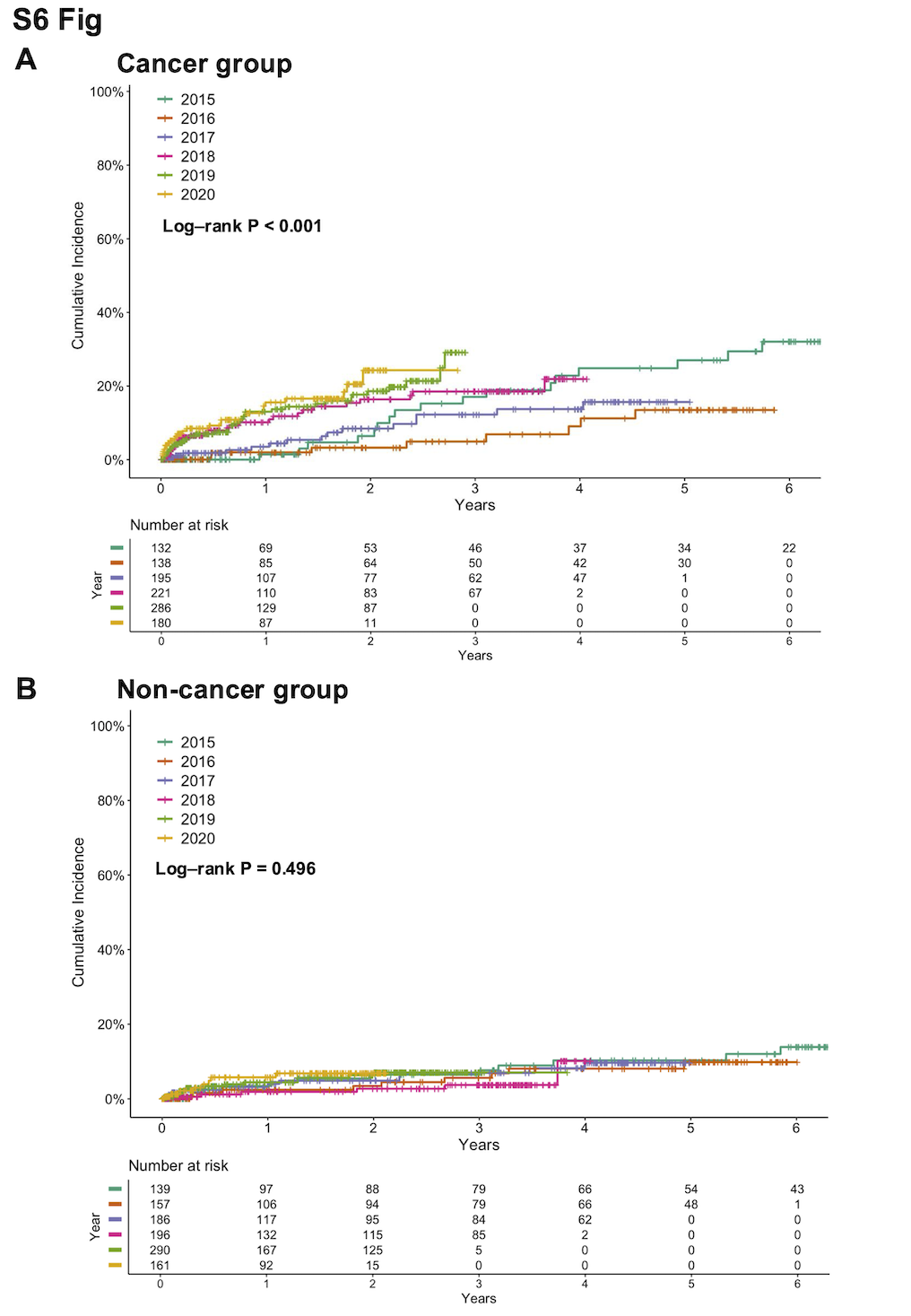

Supplement: S6 Fig — Cancer (A) and non-cancer (B) groups. VTE, venous thromboembolism. (TIF) [file pone.0329025.s006.tif]

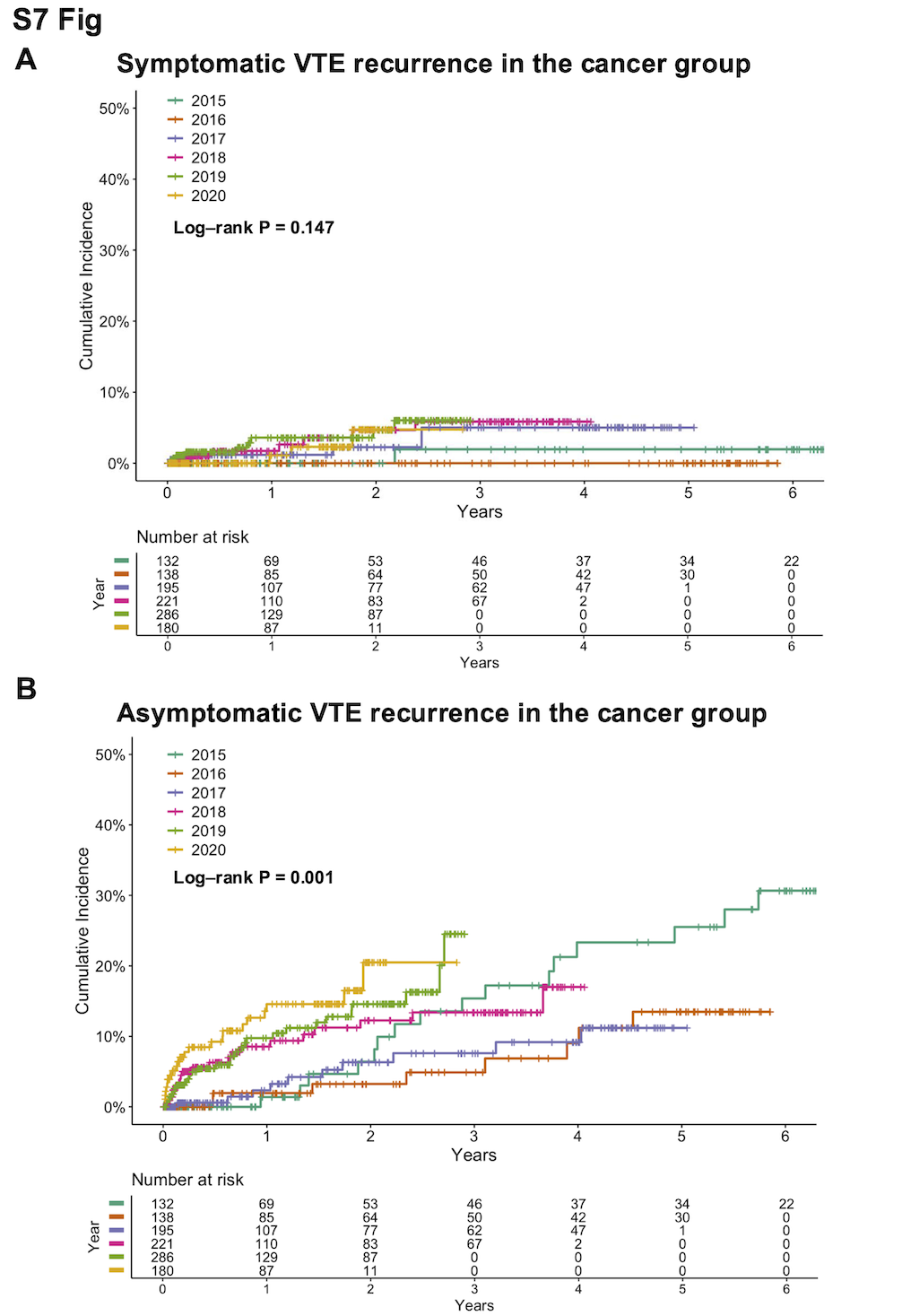

Supplement: S7 Fig — Symptomatic (A) and asymptomatic (B) VTE. VTE, venous thromboembolism. (TIF) [file pone.0329025.s007.tif]

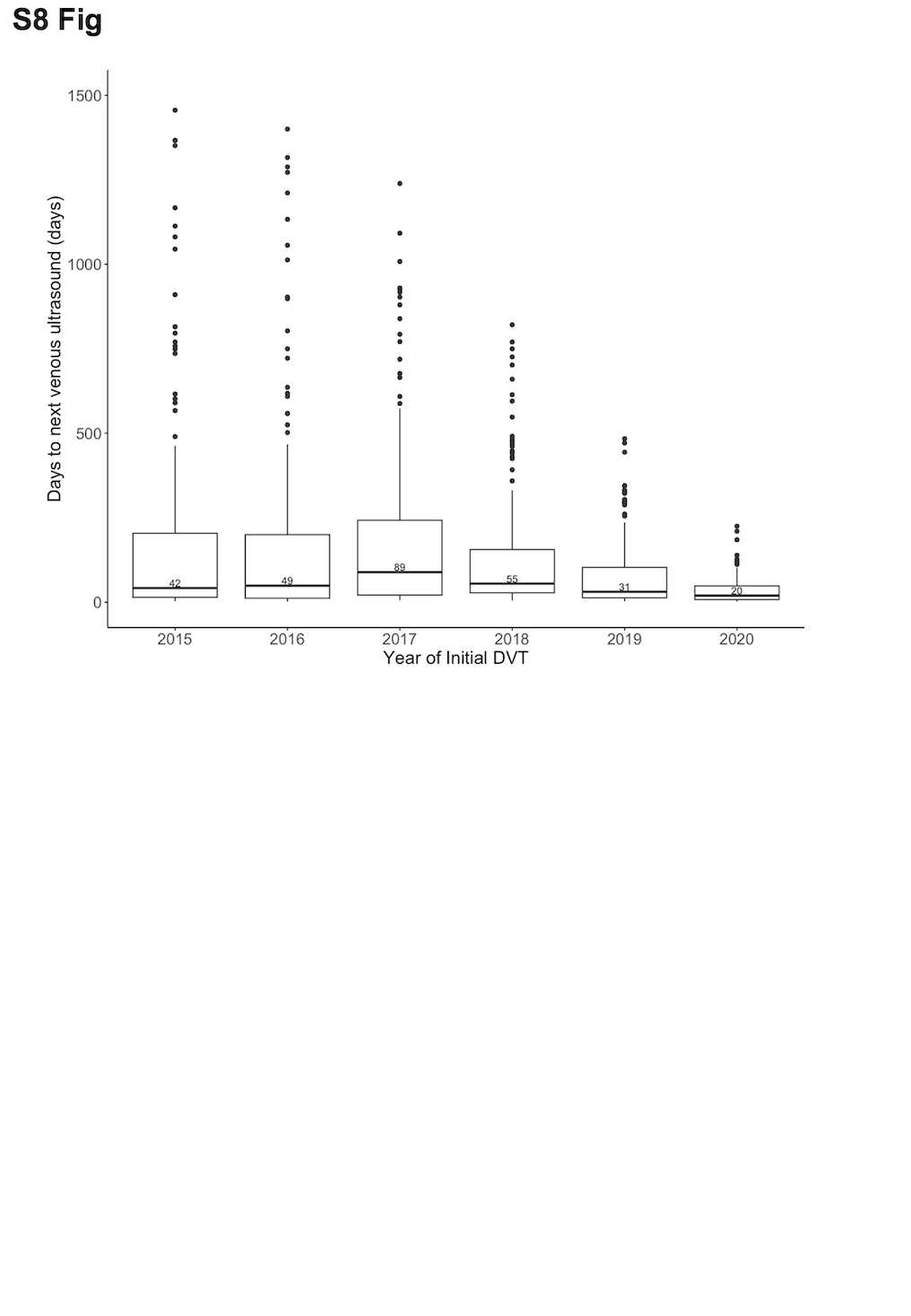

Supplement: S8 Fig — DVT, deep vein thrombosis. (TIF) [file pone.0329025.s008.tif]

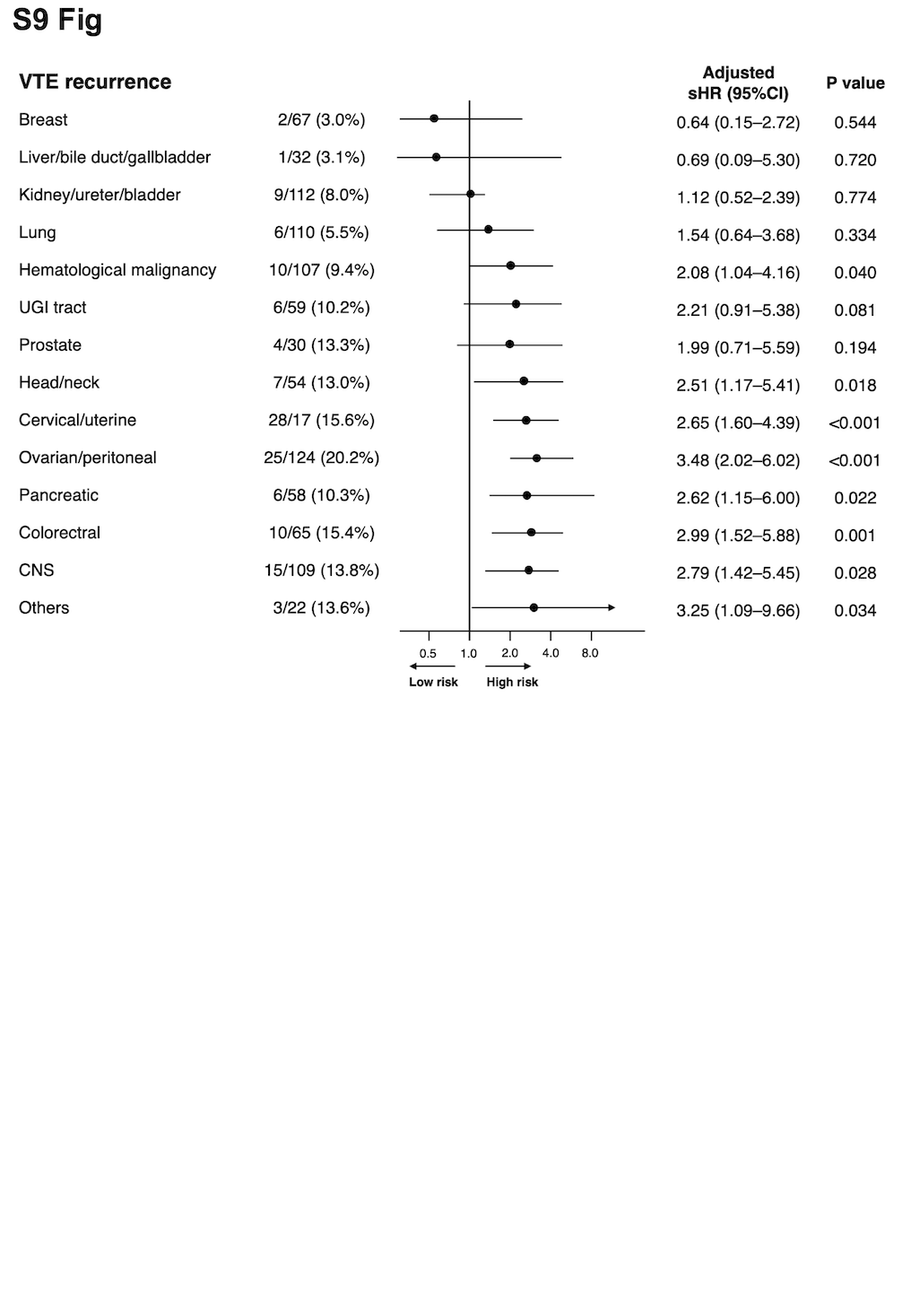

Supplement: S9 Fig — The Fine–Gray models were adjusted for age, sex, body mass index, comorbidities as a whole, baseline pulmonary embolism, hemoglobin level, anticoagulation beyond the acute phase, and discontinuation of anticoagulants. CI, confidence interval; CNS, central nervous system; sHR, subdistribution hazard ratio; UGI, upper gastrointestinal; VTE, venous thromboembolism. (TIF) [file pone.0329025.s009.tif]

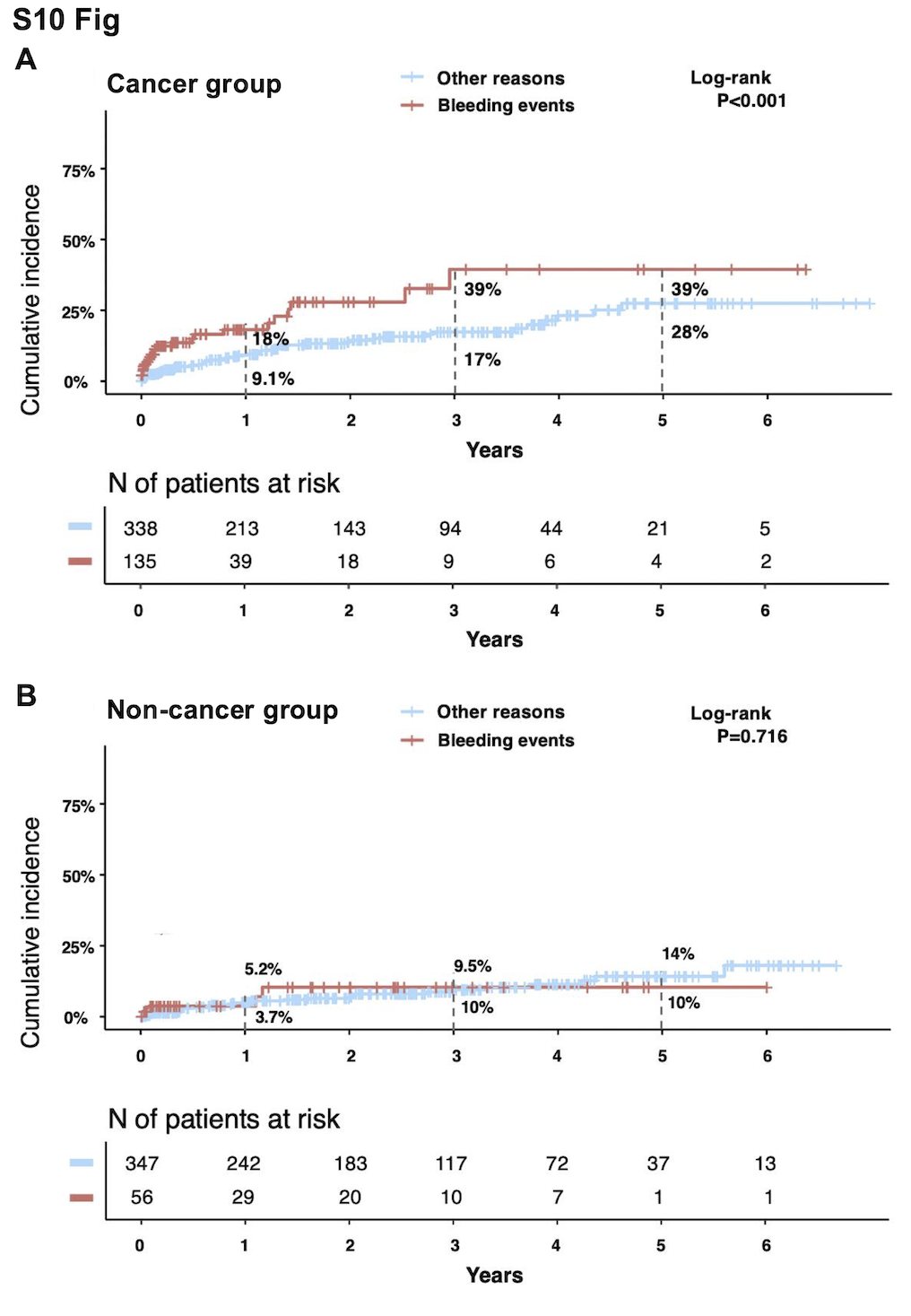

Supplement: S10 Fig — Cancer (A) and non-cancer (B) groups. VTE, venous thromboembolism. (TIF) [file pone.0329025.s010.tif]

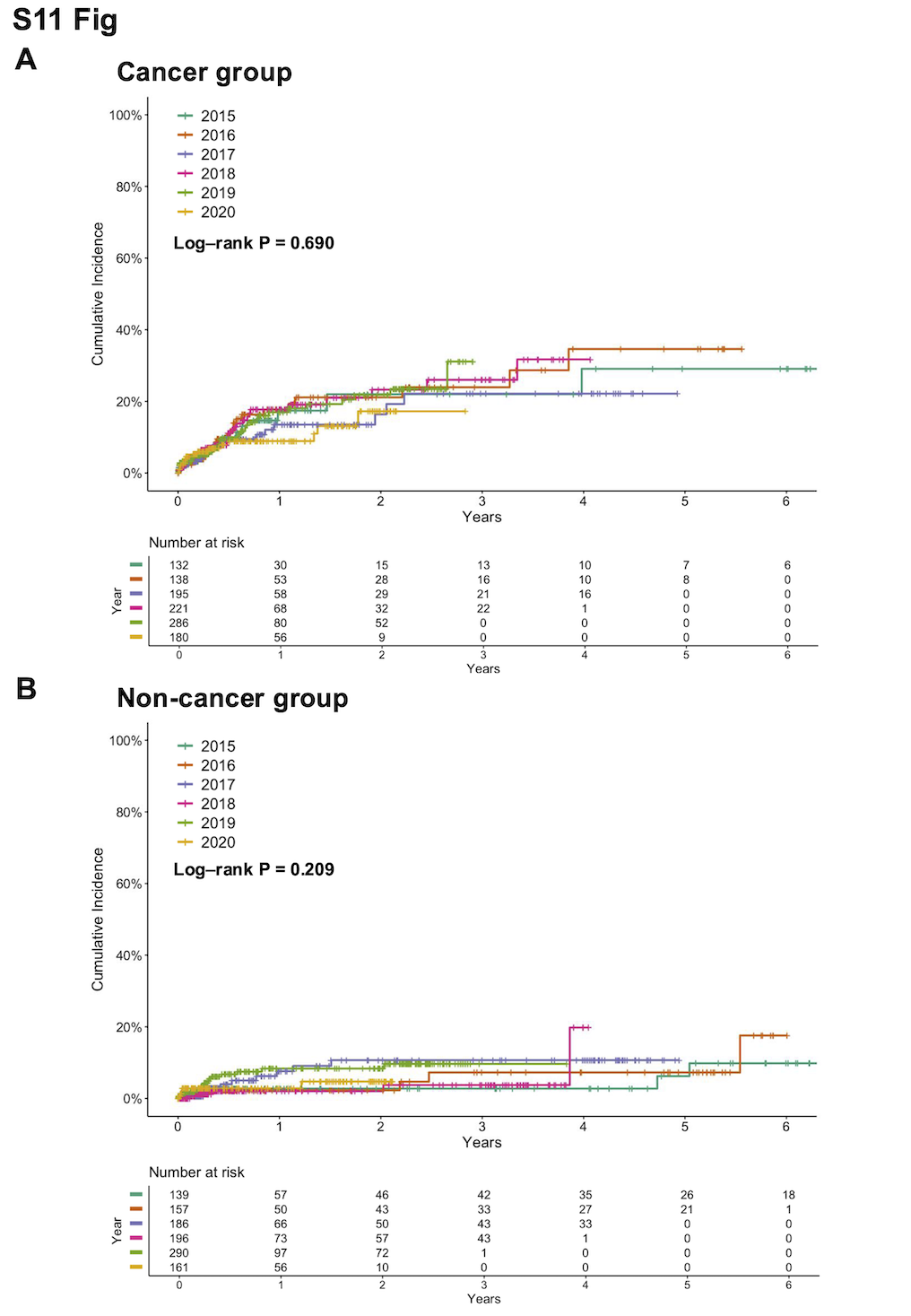

Supplement: S11 Fig — Cancer (A) and non-cancer (B) groups. (TIF) [file pone.0329025.s011.tif]

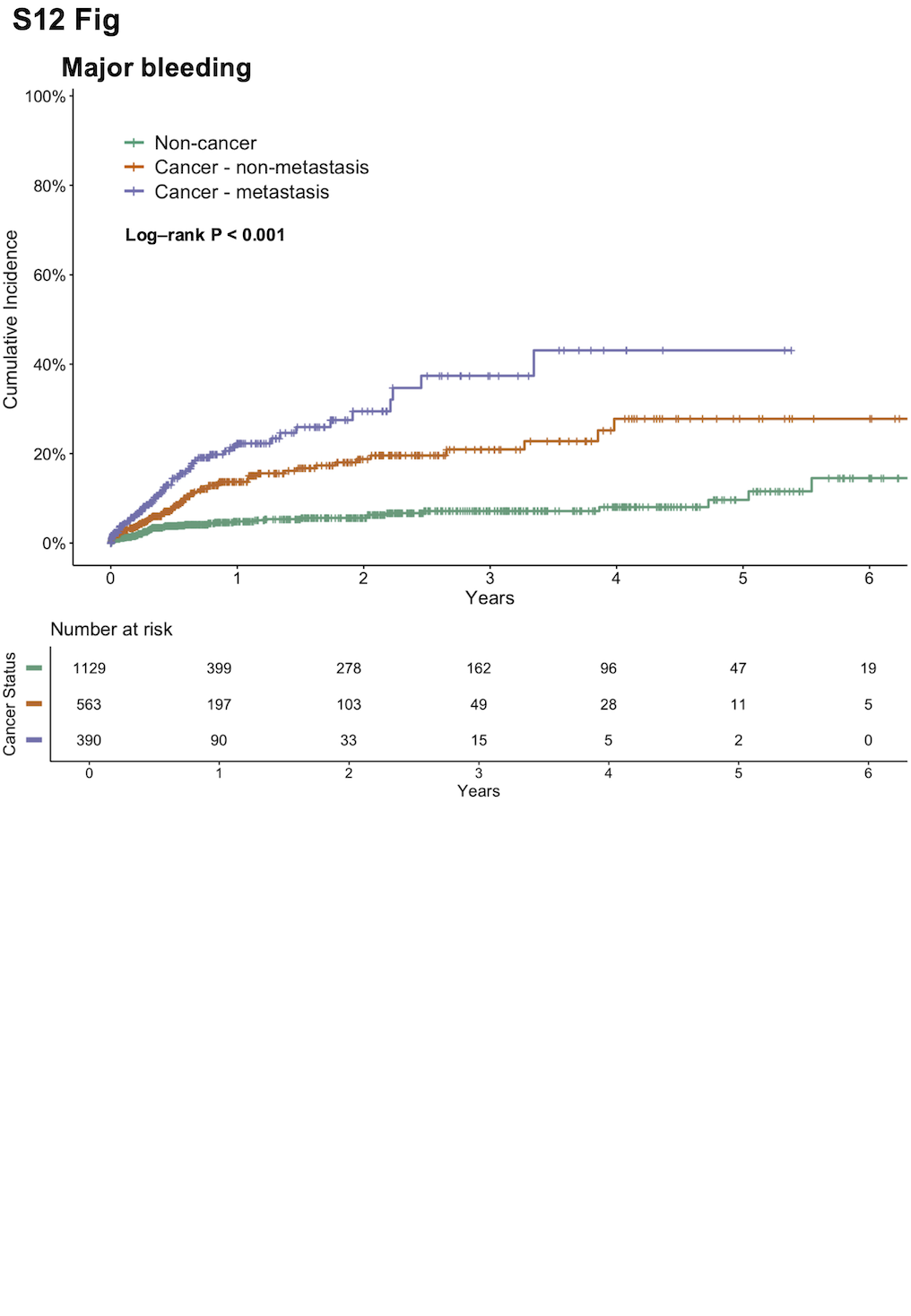

Supplement: S12 Fig — (TIF) [file pone.0329025.s012.tif]

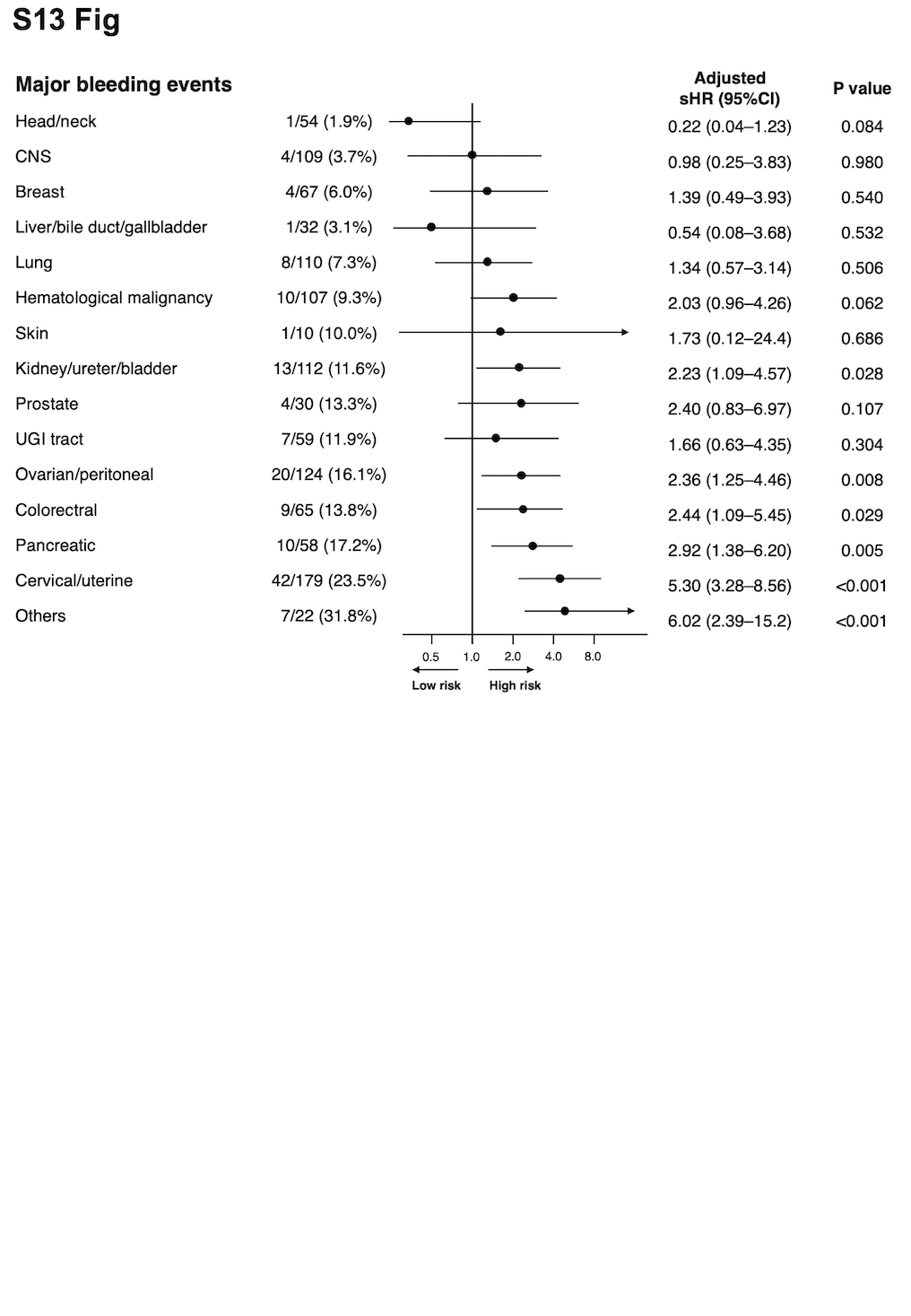

Supplement: S13 Fig — The Cox models were adjusted for age, sex, body mass index, comorbidities as a whole, baseline pulmonary embolism, hemoglobin level, and anticoagulation beyond the acute phase. CI, confidence interval; CNS, central nervous system; HR, hazard ratio; UGI, upper gastrointestinal. (TIF) [file pone.0329025.s013.tif]

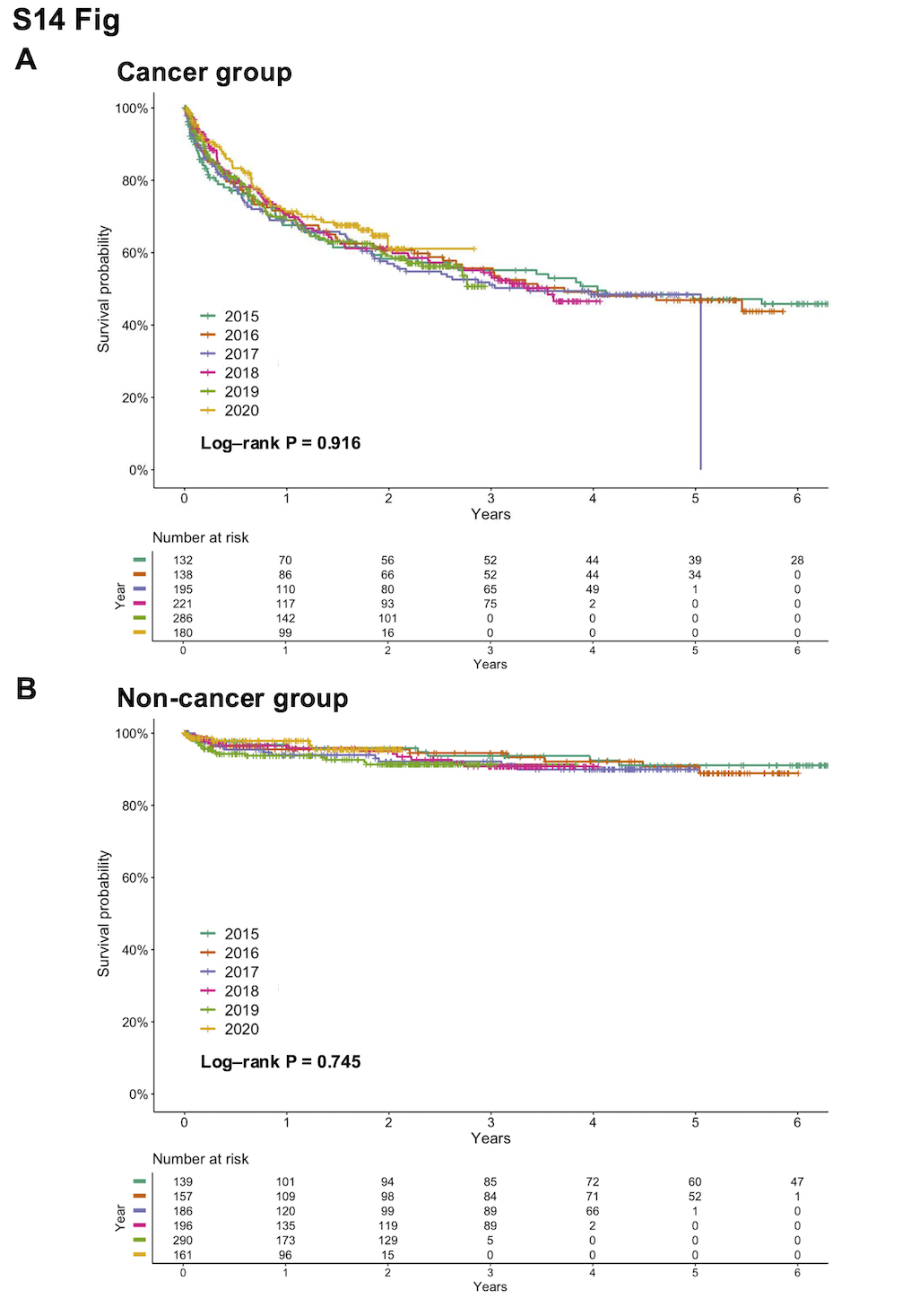

Supplement: S14 Fig — Cancer (A) and non-cancer (B) groups. (TIF) [file pone.0329025.s014.tif]

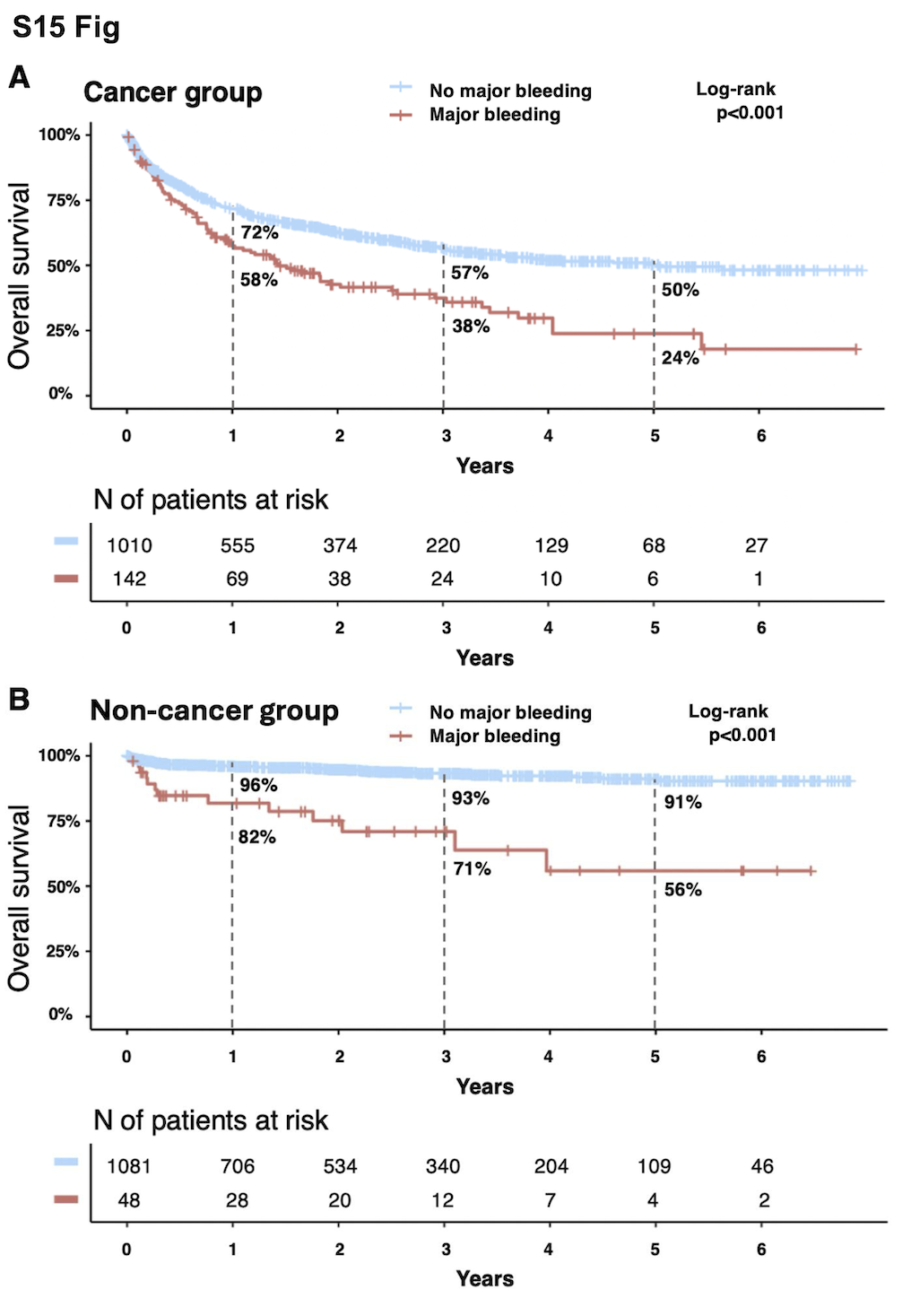

Supplement: S15 Fig — Cancer (A) and non-cancer (B) groups. (TIF) [file pone.0329025.s015.tif]

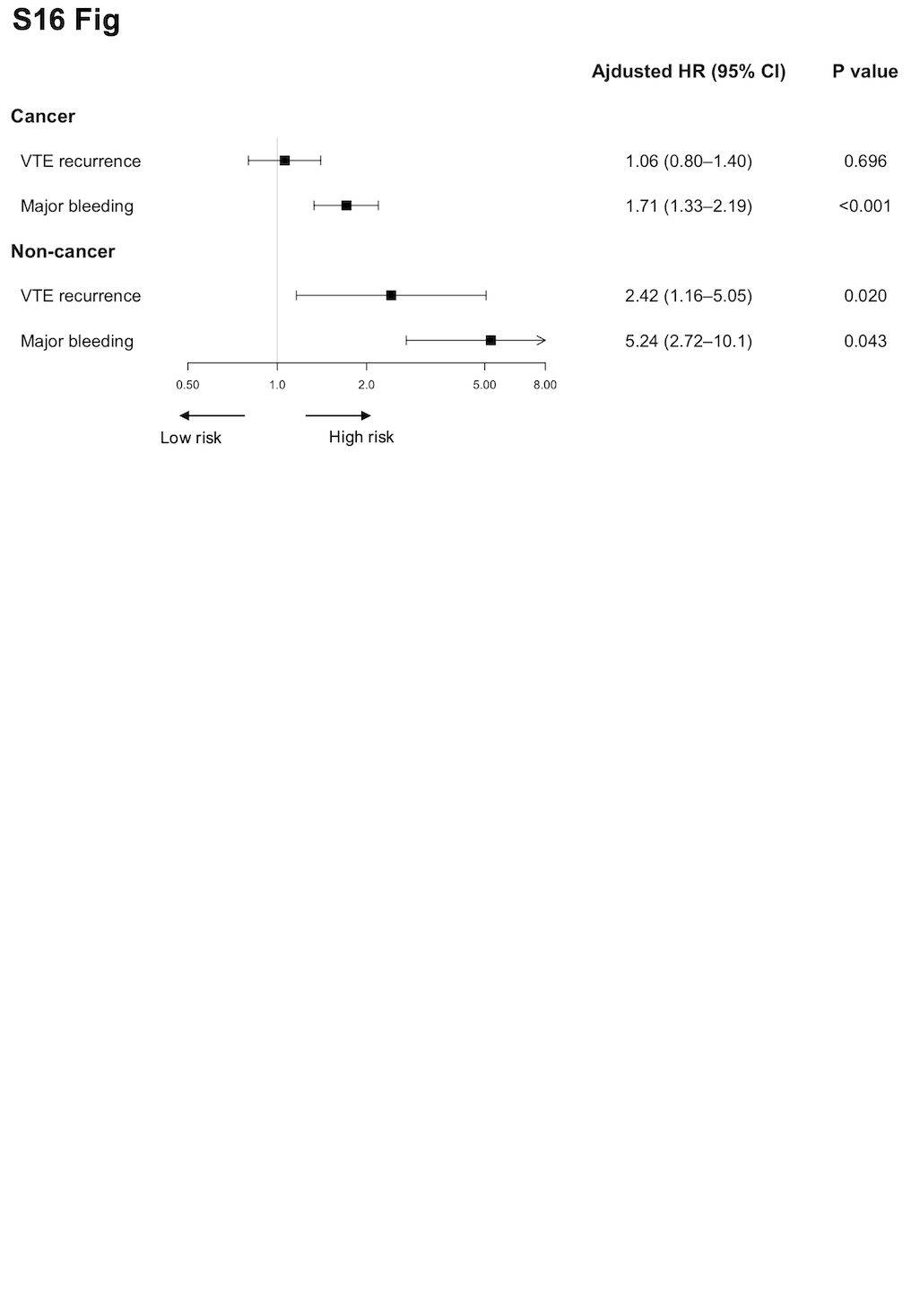

Supplement: S16 Fig — The Cox models were adjusted for age, sex, body mass index, comorbidities as a whole, baseline pulmonary embolism, hemoglobin level, anticoagulation beyond the acute phase, and discontinuation of anticoagulants, with either VTE recurrence or major bleeding included as an additional covariate in each model. CI, confidence interval; HR, hazard ratio; VTE, venous thromboembolism. (TIF) [file pone.0329025.s016.tif]

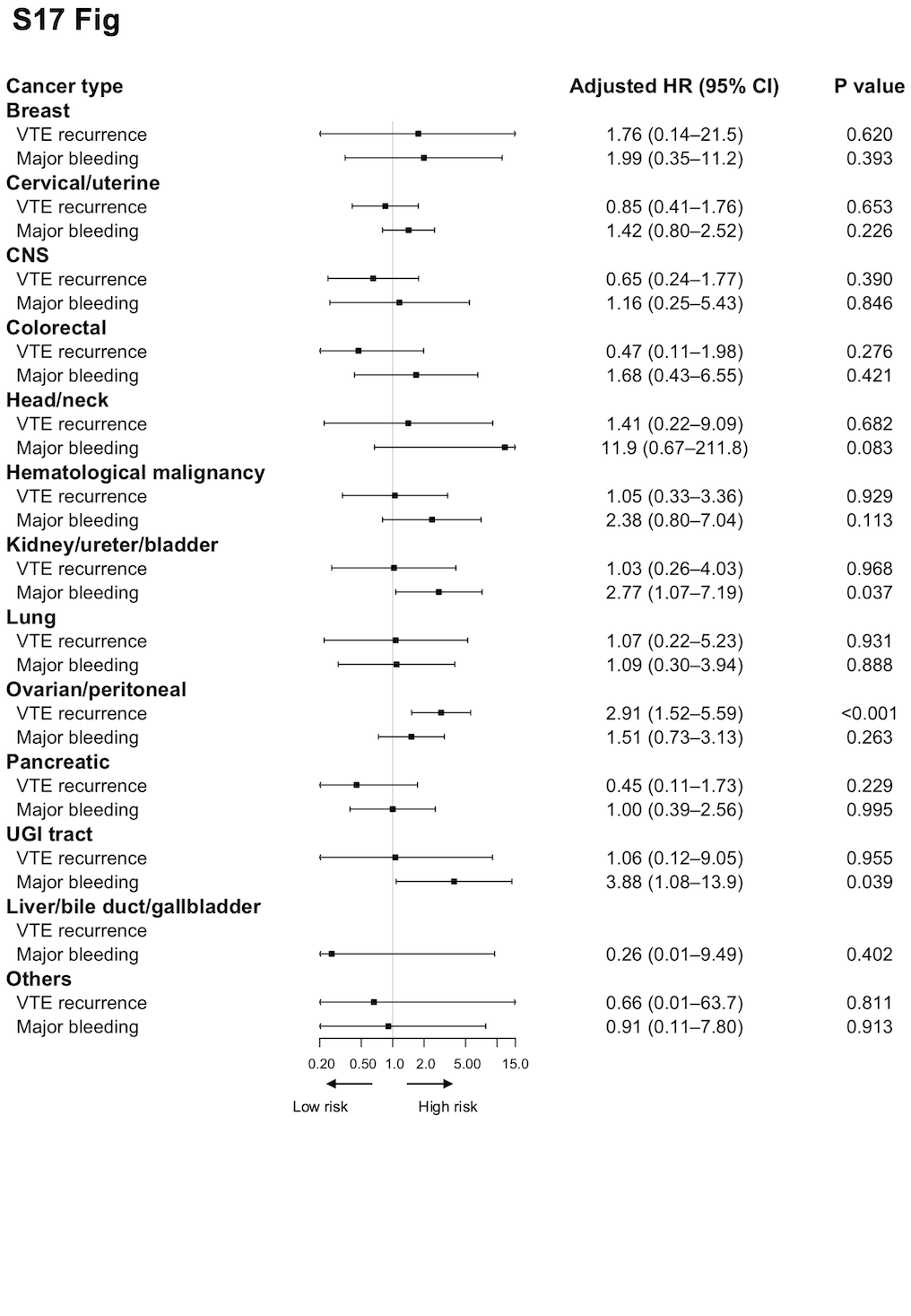

Supplement: S17 Fig — The Cox models were adjusted for age, sex, body mass index, comorbidities as a whole, baseline pulmonary embolism, hemoglobin level, anticoagulation beyond the acute phase, and discontinuation of anticoagulants, with either VTE recurrence or major bleeding included as an additional covariate in each model. CI, confidence interval; CNS, central nervous system; HR, hazard ratio; UGI, upper gastrointestinal; VTE, venous thromboembolism. (TIF) [file pone.0329025.s017.tif]
