# Supplementary material for: Risk of recurrence and bleeding in patients with cancer-associated venous thromboembolism in the direct oral anticoagulants era: Findings from the TULIPE registry
Source: PLoS One. 2025 Jul 29;20(7):e0329025. doi: 10.1371/journal.pone.0329025 (PMC12306744; doi:10.1371/journal.pone.0329025)
Supplement: Attachment — Submitted filename: 250602_Response_to_Reviewers.docx [file pone.0329025.s019.docx]

**Dear Editors and Reviewers**

We sincerely appreciate the time and effort spent reviewing and providing valuable comments on our manuscript. We are grateful to the reviewers for their favorable comments and valuable suggestions, which have helped us significantly improve our manuscript. As indicated in the following responses, we have carefully considered all the comments and suggestions in the revised version of our paper. These changes are highlighted in yellow in the revised manuscript. We have incorporated all the suggestions and believe that they have significantly improved the scientific content of the manuscript. Thank you once again.

**Response to the Academic Editor**
***Major comment #1:***
*The temporal trends in anticoagulation use or outcomes over the study period (2015–2020) are not examined. For example, did DOAC adoption increase over time, and were outcomes better in more recent years?*

**Reply:**

As suggested, we have analyzed the temporal trends in anticoagulant use during the study period. DOAC use increased from 2015 to 2020 in both the cancer and non-cancer groups (S1A Fig and S1B Fig), whereas the use of warfarin and other anticoagulants declined during the same period. We have added this information to the “Treatment strategies” section, as follows:

The trends in anticoagulant use during the study period are presented in S1 Fig. In both the cancer and non-cancer groups, the percentage of DOAC prescriptions increased over time, whereas the percentage of prescriptions for warfarin and other anticoagulants decreased. (page 13, lines 203-205)

We also performed Kaplan–Meier analyses to evaluate the temporal trends in the discontinuation of anticoagulation therapy, VTE recurrence, major bleeding, and overall survival. There were no significant differences in the rate of anticoagulation discontinuation across calendar years in either group (S3 Fig). We have added this information to the “Treatment strategies” section of the revised manuscript as follows:

There was no significant difference in the annual rate of anticoagulation therapy discontinuation during the study period. (S3 Fig). (page 14, lines 209-210)

We conducted Kaplan–Meier analyses of VTE recurrence by year (S6 Fig). In the cancer group, the cumulative incidence of VTE recurrence varied significantly by year, with notably higher recurrence rates observed in the latter part of the study period (2019–2020). No significant differences were observed in the VTE recurrence by year in the non-cancer group. To explore the factors contributing to these annual differences within the cancer group, we conducted Kaplan–Meier analyses, stratifying cases into symptomatic and asymptomatic VTE recurrences (S7 Fig). There was no significant difference in symptomatic VTE recurrence by year (S7A Fig); however, there were significant differences in asymptomatic VTE recurrence, with a marked increase from 2019 to 2020 (S7B Fig).

The precise reason for the increased recurrence of asymptomatic VTE during the latter part of the study period remains unclear. One plausible explanation is a change in follow-up practices after DVT diagnosis, leading to the more frequent use of lower-limb ultrasonography at earlier time points. S7 Fig shows a comparison of the number of days from the initial DVT diagnosis to the first post-diagnosis ultrasonography evaluation annually. The median interval was notably shortened in 2019 and 2020, suggesting that earlier follow-up may have contributed to the increased detection of asymptomatic VTE.

However, the exact reason for these shorter follow-up intervals remains unclear and is a limitation of our retrospective, single-center study. Changes in hospital policies or practices among physicians or departments may have influenced the results. These findings have been added to the “Results” section of the revised manuscript and the “Limitations” section has been expanded accordingly.

We evaluated the year-by-year trends in VTE recurrence (S6 Fig). In the cancer group, the cumulative incidence of VTE recurrence varied significantly by year, with notably higher recurrence rates observed in the latter part of the study period (2019–2020). In contrast, no significant differences in VTE recurrence by year were observed in the non-cancer group. To explore the factors contributing to these annual differences within the cancer group, we conducted Kaplan–Meier analyses, stratifying cases into symptomatic and asymptomatic VTE recurrences (S7 Fig). The results showed no significant variation by year in symptomatic VTE recurrence (S7A Fig); however, significant differences were noted in asymptomatic VTE recurrence, with a marked increase from 2019 to 2020 (S7B Fig). The precise reason for the increase in asymptomatic VTE recurrence during the latter part of the study period remains unclear. One plausible explanation is the change in follow-up practices after DVT diagnosis, leading to more frequent use of lower limb ultrasonography at earlier time points. S8 Fig compares the number of days from the initial DVT diagnosis to the first post-diagnosis ultrasonography, which was evaluated annually. The median interval was notably shortened in 2019 and 2020, suggesting that earlier follow-up may have contributed to the increased detection of asymptomatic VTE in these years. (pages 15-16, lines 226-240)

Furthermore, owing to the retrospective design, follow-up practices after the initial DVT diagnosis were at the discretion of individual physicians, and no standardized protocol was employed, which may have influenced the outcomes of this study. (pages 30-31, lines 506-508)

We also evaluated the Kaplan–Meier curves for major bleeding by year and found no temporal differences between the groups (S11 Fig). We have added this information to the “Major Bleeding” section of the revised manuscript as follows:

No temporal differences were observed in major bleeding events between the groups (S11 Fig). (page 19, lines 286-287)

We additionally evaluated the Kaplan–Meier curves for overall survival by year and found no temporal differences in either group (S14 Fig). We have added this information to the “Overall Survival” section and “Supporting information” in the revised manuscript as follows:

We also evaluated year-by-year trends in OS and found no temporal differences in either group (S14 Fig). (page 21, lines 333-334)

***Major comment #2:***
*The authors do not analyse the role of cancer stage or treatment type (e.g., chemotherapy vs. radiation) in VTE recurrence or bleeding risks. These factors likely contribute to the observed differences in outcomes.*

**Reply:**

We agree that the cancer stage and treatment type are important factors that may influence the risk of VTE recurrence and bleeding. Unfortunately, our dataset did not include information on cancer stage; however, it did include data on the presence or absence of distant metastases at the time of DVT diagnosis. We further stratified the patients into three groups—non-cancer, cancer without distant metastasis, and cancer with distant metastasis—to explore the differences in clinical outcomes. Kaplan–Meier curves were constructed to compare the cumulative incidence of VTE recurrence and major bleeding among the three groups. We found that VTE recurrence was lowest in the non-cancer group, with no significant differences between the cancer with and without metastasis groups (S5 Fig). However, major bleeding events occurred most frequently in patients with cancer and distant metastasis, followed by patients with cancer without metastasis and patients without cancer (S12 Fig). We have added this information to the “Results” section as follows:

Among the patients with cancer, 33.9% had distant metastases. (page 12, line 191)

We further stratified the patients into three groups—non-cancer, cancer without distant metastasis, and cancer with distant metastasis—to explore the differences in clinical outcomes. We found that VTE recurrence was lowest in the non-cancer group, with no significant differences between the cancer groups with and without metastasis (S5 Fig). (page 15, lines 222-226)

We further stratified the patients into three groups—non-cancer, cancer without distant metastasis, and cancer with distant metastasis—to explore differences in clinical outcomes. Major bleeding events occurred most frequently in patients with cancer with distant metastasis, followed by patients with cancer without metastasis and patients without cancer (S12 Fig). (page 19, lines 287-291)

Unfortunately, our registry did not comprehensively capture specific treatment types (e.g., chemotherapy or radiotherapy). Consequently, stratified or adjusted analyses based on these variables could not be performed. We have added this limitation to the “Limitations” section, as follows:

Finally, although we collected information on cancer type and the presence or absence of distant metastasis at baseline, we did not have access to detailed information regarding cancer stage or treatment. Consequently, we were unable to assess the influence of these factors on clinical outcomes. (page 31, lines 517-520)

***Major comment #3:***
*The subgroup analyses of cancer type lack adjustments for multiple comparisons. This undermines the reliability of some findings, such as the higher recurrence risk in CNS cancer patients.*

**Reply:**

As you correctly pointed out, the initial subgroup analyses by cancer type were not adjusted for multiple comparisons, which may have increased the risk of type I errors. In response, we reanalyzed the data using multivariate Fine-Gray models to adjust for potential confounders. For VTE recurrence, the model was adjusted for age, sex, body mass index, comorbidities as a whole, baseline pulmonary embolism, hemoglobin level, anticoagulation beyond the acute phase, and discontinuation of anticoagulants (S9 Fig). For major bleeding, the model was similarly adjusted, excluding the discontinuation of anticoagulants (S13 Fig). Based on these analyses, the descriptions in the revised manuscript have been updated as follows:

Further stratification by cancer type revealed that hematological malignancies, head/neck, cervical/uterine, ovarian/peritoneal, pancreatic, colorectal, and CNS cancers were significantly associated with a higher risk of VTE recurrence (S9 Fig). (page 18, lines 266-268)

Further stratification according to cancer type revealed that pancreatic, cervical/uterine, colorectal, kidney/ureter/bladder, and ovarian/peritoneal cancers were associated with a higher risk of major bleeding (S13 Fig). (page 21, lines 318-320)

Additionally, details of the analytical methods and covariates are provided in the “Statistical Analysis” section, as follows:

Given the potential impact of mortality as a competing risk, the Fine–Gray subdistribution hazard model was applied to estimate the subdistribution hazard ratios (sHRs) for VTE recurrence and major bleeding. In these models, death from other causes was treated as a competing event, whereas in the Cox model, it was treated as a censored event for comparison. Multivariate models were adjusted for age, sex, BMI, comorbidities as a whole, baseline PE, hemoglobin level, anticoagulation beyond the acute phase, and discontinuation of anticoagulant therapy. Notably, discontinuation was excluded from the adjustment in the major bleeding model because bleeding events occurring after discontinuation were not systematically captured in our registry. Subgroup analyses for VTE recurrence and major bleeding were conducted using Fine–Gray subdistribution hazard models, adjusting for the same covariates in the primary analyses. To evaluate the impact of VTE recurrence and major bleeding on OS, additional subgroup analyses were performed using Cox proportional hazards models, incorporating VTE recurrence or major bleeding as covariates along with the previously described variables. (page 9, lines 144-157)

***Major comment #4:***
*The survival analyses focus primarily on differences between cancer and non-cancer groups but do not explore predictors of survival within the cancer group. For example, how does the presence of major bleeding or VTE recurrence impact survival in different cancer subtypes?*

**Reply:**

We conducted multivariate analyses using the Cox proportional hazards model to evaluate the impact of VTE recurrence and major bleeding on overall survival in patients with and without cancer (S16 Fig). Among the cancer group, major bleeding was significantly associated with poorer survival (adjusted HR: 1.71; 95% CI: 1.33–2.19; P < 0.001), whereas VTE recurrence was not (adjusted HR: 1.06; 95% CI: 0.80–1.40; P = 0.696). In contrast, in the non-cancer group, both VTE recurrence (adjusted HR: 2.42; 95% CI: 1.16–5.05; P = 0.020) and major bleeding (adjusted HR: 5.24; 95% CI: 2.72–10.1; P = 0.043) were significantly associated with worse survival. Furthermore, we performed stratified analyses according to cancer type (S17 Fig). We found that VTE recurrence was significantly associated with decreased survival in ovarian/peritoneal cancer (adjusted HR: 2.91; 95% CI: 1.52–5.59; P < 0.001), while major bleeding was associated with poorer survival in kidney/ureter/bladder cancer (adjusted HR: 2.77; 95% CI: 1.07–7.19; P = 0.037), and upper gastrointestinal tract cancers (adjusted HR: 3.88; 95% CI: 1.08–13.9; P = 0.039). These results have been included in the "Overall survival" subsection of the Results section as follows:

We further investigated the impact of VTE recurrence and major bleeding on the OS of patients with and without cancer (S16 Fig). Among the cancer group, major bleeding was significantly associated with poorer survival (adjusted HR: 1.71; 95% CI: 1.33–2.19; P < 0.001), whereas VTE recurrence was not (adjusted HR: 1.06; 95% CI: 0.80–1.40; P = 0.696). In contrast, in the non-cancer group, both VTE recurrence (adjusted HR: 2.42; 95% CI: 1.16–5.05; P = 0.020), and major bleeding (adjusted HR: 5.24; 95% CI: 2.72–10.1; P = 0.043) were significantly associated with worse survival. Furthermore, we performed stratified analyses according to the cancer type. VTE recurrence was significantly associated with decreased survival in ovarian/peritoneal cancer (adjusted HR: 2.91; 95% CI: 1.52–5.59; P < 0.001), and major bleeding was strongly associated with poorer survival in kidney/ureter/bladder cancer (adjusted HR: 2.77; 95% CI: 1.07–7.19; P = 0.037), and upper gastrointestinal tract cancer (adjusted HR: 3.88; 95% CI: 1.08–13.9; P = 0.039) (S17 Fig). (pages 22-23, lines 346-357)

***Specific comment #1:***

*In the abstract, details on subgroup findings or the implications of anticoagulation discontinuation, which are important outcomes, are lacking. Please include.*

**Reply:**

As suggested, we have revised the Abstract to include the impact of anticoagulation discontinuation on VTE recurrence, which is one of the key findings of our study, as follows:

In the cancer group, discontinuation of bleeding-related anticoagulation therapy was associated with increased VTE recurrence (P < 0.001), whereas no such association was observed in the non-cancer group (P = 0.716). (page 3, lines 38-41)

***Specific comment #2:***

*The introduction does not clearly differentiate how the study builds upon existing literature, such as the COMMAND VTE Registry studies. The rationale for focusing on cancer patients with DVT diagnosed via ultrasound is not sufficiently justified. A discussion of why asymptomatic cases are important to study would provide more clarity.*

**Reply:**

As suggested, we have revised the Introduction section to clarify how our study differs from previous reports, such as the COMMAND VTE Registry (Sakamoto J, et al. *Circ J* 2019, PMID: 31548438) and COMMAND VTE Registry-2 (Chatani R, et al. *Thromb Res* 2024, PMID: 38190788), which primarily included patients with symptomatic VTE. Our study included both symptomatic and asymptomatic VTE cases, reflecting real-world cancer care. We have also cited recent studies suggesting that asymptomatic VTE may be associated with recurrence and poor prognosis in patients with cancer. This revision has been added to the “Introduction” section as follows:

In particular, existing studies, such as the COMMAND VTE Registry and COMMAND VTE Registry-2, primarily focused on symptomatic VTE and did not include asymptomatic DVT [6-7], which is increasingly detected through routine ultrasonography in patients with cancer. However, previous studies have shown that asymptomatic DVT is associated with poor survival in ambulatory patients with cancer [8], and even asymptomatic isolated distal DVT carries a non-negligible risk of recurrence, especially after discontinuation of anticoagulation therapy [9]. These findings underscore the clinical importance of asymptomatic DVT in the field of oncology. Therefore, it is essential to evaluate both symptomatic and asymptomatic DVT in real-world practice to better understand its prognostic implications and inform anticoagulation decisions in patients with cancer.

This study aimed to evaluate the clinical characteristics and long-term outcomes of patients with cancer- and non-cancer-associated VTE in real-world settings, including both symptomatic and asymptomatic patients. (pages 4-5, lines 59-71)

***Specific comment #3:***

*The study excludes patients with DVT diagnosed before 2015 to focus on the DOAC era but does not clarify whether warfarin use in 9.4% of the cohort is representative of contemporary practice. This could bias the findings. Please explain.*

**Reply:**

Thank you for pointing this out. Although our study focused on the DOAC era, 9.4% of the patients were still treated with warfarin. Notably, this proportion is consistent with real-world data from the COMMAND VTE Registry-2, which also focused on the DOAC era (2015–2020), reported that 10.9% of cancer patients with VTE received warfarin (Chatani et al., *Thromb Res* 2024. PMID: 38190788). Therefore, we believe that the anticoagulation patterns in our cohort accurately reflects real-world practices during this period. We added this point to the revised “Discussion” section as follows:

Although our study focused on the DOAC era, 9.4% of the patients were still treated with warfarin. Notably, this proportion is consistent with real-world data from the COMMAND VTE Registry-2, which reported that 10.9% of patients with cancer-associated VTE received warfarin during the same period (2015–2020) [7]. Therefore, we believe that our treatment distribution accurately reflects the contemporary prescription patterns in Japan. However, the inclusion of a minority of warfarin users may limit the generalizability of our findings to a purely DOAC–treated population. In this context, our results support the view that patients with cancer remain at an increased risk of VTE recurrence, regardless of the anticoagulant type. (page 25, lines 393-401)

***Specific comment #4:***

*While the Fine-Gray subdistribution hazard model accounts for competing risks, the authors does not report sensitivity analyses to test the robustness of its findings. This is critical given the high mortality rate in the cancer group.*

**Reply:**

Thank you for your comments. To assess the robustness of our findings from the Fine–Gray subdistribution hazard model, we conducted sensitivity analyses using Cox proportional hazards models for both VTE recurrence and major bleeding, treating death as a censoring event. These analyses confirmed that cancer remained significantly associated with both outcomes, which is consistent with the results of the Fine–Gray model. We have added this information to the “Statistical Analysis” section and the Results sections for “VTE recurrence” and “Major bleeding,” as follows:

Given the potential impact of mortality as a competing risk, the Fine–Gray subdistribution hazard model was applied to estimate the subdistribution hazard ratios (sHRs) for VTE recurrence and major bleeding. In these models, death from other causes was treated as a competing event, whereas in the Cox model, it was treated as a censored event for comparison. (page 9, lines 144-148)

For the sensitivity analysis, we applied a Cox proportional hazards model to treat death as a censored event. Cancer remained significantly associated with VTE recurrence (adjusted HR: 2.47; 95% CI: 1.78–3.42; P < 0.001), supporting the robustness of the findings obtained from the Fine–Gray model. (page 16, lines 245-248)

For sensitivity analysis, we applied a Cox proportional hazards model to treat death as a censored event. Cancer remained significantly associated with major bleeding risk (adjusted HR: 2.71; 95% CI: 1.52–3.83; P < 0.001), supporting the results obtained from the Fine–Gray model. (page 19, lines 300-302)

***Specific comment #5:***

*The study does not mention whether validated bleeding risk scores were used to adjust for bleeding risk in the cancer cohort. This could affect the accuracy of the hazard ratios.*

**Reply:**

Unfortunately, validated bleeding risk scores such as HAS-BLED or RIETE were not used in this study. This was primarily because of the retrospective nature of the registry and the lack of complete data required to calculate such scores. We have added this information to the “Discussion” section, as follows:

However, in our retrospective study, the application of validated bleeding risk scores, such as the HAS-BLED, was not feasible owing to missing data. (pages 26-27, lines 430-432)

***Specific comment #6:***

*Subgroups based on cancer type are analyzed but not adjusted for multiple comparisons. This raises the risk of type I error. This should be addressed.*

**Reply:**

Thank you for your insightful comment. As you have correctly pointed out, the initial subgroup analyses by cancer type were not adjusted for multiple comparisons, which could have increased the risk of a type I error. In response, we reanalyzed the data using multivariate Fine-Grey models to adjust for potential confounders. For VTE recurrence, the model included age, sex, body mass index, comorbidities as a whole, baseline pulmonary embolism, hemoglobin level, anticoagulation beyond the acute phase, and discontinuation of anticoagulants (S10 Fig). For major bleeding, the same model was used, excluding the discontinuation of anticoagulants (S14 Fig). We have revised the manuscript accordingly and added the following statement to the “VTE recurrence” section and “Major bleeding” sections, as follows:

Further stratification by cancer type revealed that hematological malignancies, head/neck, cervical/uterine, ovarian/peritoneal, pancreatic, colorectal, and CNS cancers were significantly associated with a higher risk of VTE recurrence (S9 Fig). (page 18, lines 266-268)

Further stratification according to cancer type revealed that pancreatic, cervical/uterine, colorectal, kidney/ureter/bladder, and ovarian/peritoneal cancers were associated with a higher risk of major bleeding (S13 Fig). (page 21, lines 318-320)

***Specific comment #7:***

*Table 1: While the baseline characteristics are comprehensive, the authors do not report p-values for all comparisons, such as DVT location. Any non-significant findings should still be reported.*

**Reply:**

Thank you for pointing this out. We have revised Table 1 to include the p-values for all baseline comparisons, including variables such as DVT location.

***Specific comment #8:***

*Table 4: The all-cause mortality data is significant, but the breakdown of causes of death is underexplored in the discussion. For example, the higher rate of cancer-related deaths in the cancer group is expected, but the significance of fatal bleeding events in cancer patients warrants further elaboration.*

**Reply:**

The clinical details of the six fatal bleeding events that occurred in the cancer group were as follows: intracranial hemorrhage (n=2), gastrointestinal bleeding (n=2), tumor-associated intraperitoneal bleeding (n=1), and traumatic head injury (n=1). We have added this information in the “Results” section. Furthermore, we revised the “Discussion” section to highlight the clinical relevance of these fatal events and their implications for the anticoagulation management of cancer-associated thrombosis. We have added the following statements to the manuscript:

Notably, six fatal bleeding events occurred in the active cancer group, whereas none occurred in the non-cancer group. These included intracranial hemorrhage (n = 2), gastrointestinal bleeding (n = 2), tumor-associated intraperitoneal bleeding (n = 1), and traumatic head injury (n = 1); all patients receiving anticoagulation at the time of death. (pages 21-22, lines 336-339)

Although cancer-related deaths accounted for the majority of mortalities in the active cancer group, six patients experienced fatal bleeding during anticoagulation therapy. These included intracranial hemorrhage, gastrointestinal bleeding, and tumor-associated vascular rupture. These findings highlight the complex clinical dilemma of balancing thrombosis and bleeding risks in patients with advanced malignancy and underscore the importance of individualized anticoagulation strategies in this vulnerable population. (page 25, lines 405-410)

***Specific comment #9:***

*Figure 3 and 5: Subgroup analyses are presented without adjustments for multiple comparisons. The authors should clarify this limitation in the results section.*

**Reply:**

We appreciate the reviewer’s comment. We have revised the subgroup analyses in Figs 3 and 5 using multivariable Fine–Gray models adjusted for clinically relevant covariates. These models included adjustments for age, sex, body mass index (BMI), comorbidities, baseline PE, hemoglobin level, anticoagulant use beyond the acute phase, and discontinuation of anticoagulants. We have updated the manuscript accordingly and added the following statements to the “VTE recurrence” and “Major bleeding” sections, as follows:

Subgroup analyses using multivariable Fine–Gray models showed a consistent trend toward a higher risk of VTE recurrence in patients with cancer than in those without cancer (Fig 3). (pages 17-18, lines 264-266)

Subgroup analyses using multivariable Fine–Gray models showed a consistent trend toward a higher risk of major bleeding events in patients with cancer than in patients without cancer (Fig 5). (pages 20-21, lines 316-318)

***Specific comment #10:***

*The similar rates of anticoagulation discontinuation between groups are reported without sufficient discussion of the clinical implications. For example, the higher rate of discontinuation due to bleeding in the cancer group suggests a need for better bleeding risk stratification.*

**Reply:**

We have revised the Discussion section to emphasize the need for individualized bleeding risk stratification to address the clinical implications of anticoagulation discontinuation due to bleeding in patients with cancer. In particular, we highlight the importance of considering renal dysfunction, weight loss, and potential drug–drug interactions with anticancer agents that affect DOAC metabolism. We have added this information to the “Discussion” section, as follows:

This finding underscores the need for improved bleeding risk stratification in patients with cancer receiving anticoagulation therapy. Recent studies have shown that pharmacokinetic interactions between DOACs and anticancer agents exist, but most are mild, and treatment modifications are generally recommended only in clinically significant cases [30]. In contrast, certain anticancer drugs have been associated with an increased risk of bleeding when co-administered with DOACs [31], highlighting the importance of individualized assessment that considers factors such as renal dysfunction, weight loss, potential drug–drug interactions with anticancer agents, and appropriate dose adjustment in patients with cancer. (pages 27-28, lines 443-451)

***Specific comment #11:***

*The discussion does not adequately address the limitations of the study design, particularly the retrospective nature, single-center setting, and the inclusion of asymptomatic DVT cases. Besides, the potential impact of warfarin use in a minority of patients is not discussed in detail. How might this affect the generalizability of the results to a purely DOAC-treated population?*

*The discussion could expand on the implications of VTE recurrence in patients who discontinued anticoagulation due to bleeding. For instance, should alternative anticoagulants (e.g., low-dose DOACs) or non-pharmacologic strategies be considered in these patients?*

*Authors should also consider expanding on the VTE/DVT management (see Sun et al PMID: 37175686)*

**Reply:**

We have expanded the limitations section to explicitly address the retrospective design, single-center setting, and inclusion of asymptomatic DVT cases. We had added this information to the “Discussion” section, as follows:

This study had some limitations. First, as a single-center study, the prevalence of the disease may have been influenced by regional and institutional factors, potentially introducing a bias. Additionally, the retrospective nature of our study and the inclusion of both symptomatic and asymptomatic DVT cases, some of which were detected incidentally during routine ultrasonography, may have limited the generalizability of our findings to populations with acute symptomatic VTE. (page 30, lines 501-506)

We also addressed the potential impact of warfarin in 9.4% of our cohort. Although our study focused on the DOAC era, this proportion reflects real-world practices in Japan during the study period. Nevertheless, we now recognize that this may affect the generalizability of the findings to a purely DOAC-treated population and have added the following discussion accordingly:

Although our study focused on the DOAC era, 9.4% of the patients were still treated with warfarin. Notably, this proportion is consistent with real-world data from the COMMAND VTE Registry-2, which reported that 10.9% of patients with cancer-associated VTE received warfarin during the same period (2015–2020) [7]. Therefore, we believe that our treatment distribution accurately reflects the contemporary prescription patterns in Japan. However, the inclusion of a minority of warfarin users may limit the generalizability of our findings to a purely DOAC–treated population. In this context, our results support the view that patients with cancer remain at an increased risk of VTE recurrence, regardless of the anticoagulant type. (pages 24-25, lines 393-401)

We have expanded the Discussion section to address the clinical implications of VTE recurrence in patients with cancer who discontinued anticoagulation therapy due to bleeding. We now mention that alternative strategies, such as dose-reduced DOACs or non-pharmacological options, such as IVC filters, may be considered in the selection of high-risk cases. Additionally, we have cited the article suggested by the reviewer and expanded the discussion to highlight the need for individualized treatment decisions as follows:

In such cases, alternative management strategies, such as resuming anticoagulation with a reduced DOAC dose or considering non-pharmacological interventions, such as inferior vena cava filters, may be required. Clinicians should carefully consider each patient’s underlying condition, renal function, personal preferences, and lifestyle when determining the most appropriate treatment approach [35]. (pages 28, lines 461-465)

Furthermore, we have revised the final paragraph of the Discussion section to incorporate recent recommendations from the 2023 ASCO guidelines, which emphasize careful bleeding risk assessment in patients with gastrointestinal and genitourinary malignancies. We have added this information to the “Discussion” section as follows:

As outlined in the 2023 ASCO guideline, LMWH or direct oral factor Xa inhibitors are recommended for the treatment of CAT [4]. However, careful assessment of the bleeding risk is essential in patients with gastrointestinal or genitourinary malignancies. Clinicians are increasingly challenged to maintain effective anticoagulation therapy while minimizing bleeding complications in high-risk populations. (page 29, lines 473-477)

**Response to Reviewer #1**
***Comment #1:***

*Thank you very much for providing the opportunity to review the manuscript. It is written in a straight forward manner, with a clear research question, a suitable method and statistics and a clear result. There is littele to discuss or to amend. Results seems reasonable. It would have been interesting to compare the 4 DOAC among each other regarding thrombosis and bleeding. Manybe a follow-up project?*

*There are very few typos, which can be corrected upon processing.*

**Reply:**

We thank the reviewers for their positive feedback. We are grateful that you have found our research question, methodology, and results to be clearly presented and appropriate. As suggested, a comparison of the four DOAC in terms of thrombosis and bleeding risk is of great clinical interest. However, given the limited sample size and potential for confounding by indication in our current dataset, we were unable to perform a robust head-to-head analysis within this study. We agree that this will be a valuable focus of future prospective registry-based studies.

**Response to Reviewer #2**
***Comment #1:***

*Introduction:*

*Problem statement: Not clear. Please spell it out in a stand-alone paragraph.*

*Study objectives: Not clear. Please spell it out in a stand-alone paragraph. Please spell out the primary and secondary objectives.*

**Reply:**

In accordance with your suggestion, we have revised the Introduction section to clearly delineate the problem statement and study objectives. Specifically, we have structured the Introduction section to include a dedicated paragraph outlining the clinical gap regarding asymptomatic DVT in patients with cancer, which has not been sufficiently addressed in previous studies. Furthermore, we have added a standalone paragraph explicitly stating the primary and secondary objectives of the study. We have added this information to the “Introduction” section as follows:

This study aimed to evaluate the clinical characteristics and long-term outcomes of patients with cancer- and non-cancer-associated VTE in real-world settings, including both symptomatic and asymptomatic patients. The primary outcomes of this study were the incidence of VTE recurrence, major bleeding, and overall survival, each of which was compared between the active cancer and non-cancer groups. Secondary outcomes included the impact of anticoagulation discontinuation, particularly because of bleeding, VTE recurrence, major bleeding, overall survival, and cancer-type-specific risks of VTE recurrence and major bleeding. These were evaluated using multivariable Fine–Gray and Cox proportional hazards models, as appropriate. (page 5, lines 69-76)

***Comment #2:***

*Methods:*

*Line 62 – 63: The registry is a single-center, retrospective cohort study.*

*Naturally when we do a registry, we either enter data on the spot or later, some may have a follow-up. Therefore, please explain why this registry is retrospective, or otherwise.*

**Reply:**

Our registry (The TULIPE registry) is a single-center, retrospective cohort study that included consecutive patients who underwent lower-extremity venous ultrasound between September 1, 2011, and August 31, 2020, at the University of Tsukuba Hospital. Our registry study involved registering patients in the registry; however, rather than prospectively following and observing the patients over time, we performed retrospective analyses at specific points in time (January 7, 2021, and October 2, 2022) to investigate the outcomes and related factors. Nonetheless, this approach has been commonly used in similar studies, and we believe that it does not constitute a contradiction.

For example:

1. Lambertini M, Blondeaux E, Agostinetto E, Hamy AS, Kim HJ, Di Meglio A, et al. Pregnancy after breast cancer in young BRCA carriers: An international hospital-based cohort study. *JAMA*. 2024;331(1):49–57. doi:10.1001/jama.2023.25463. PMID: 38059899

2. Guo T, Fan Y, Chen M, Wu X, Zhang L, He T, et al. Cardiovascular implications of fatal outcomes of patients with coronavirus disease 2019 (COVID-19). *JAMA Cardiol*. 2020;5(7):811–8. doi:10.1001/jamacardio.2020.1017. PMID: 32219356

3. Sakamoto J, Yamashita Y, Morimoto T, Amano H, Takase T, Hiramori S, et al. Cancer-associated venous thromboembolism in the real world: From the COMMAND VTE Registry. *Circ J*. 2019;83(11):2271–81. doi:10.1253/circj.CJ-19-0515. PMID: 31548438

***Comment #3:***

*Line 74: Among the 17,188 patients enrolled in the TULIPE registry.*

*These are adult patients only or include children. If adults only, please define the cut-off age.*

**Reply:**

Our registry did not have an age limit for patient registration, and patients aged 3–101 years were included in the study. However, among these, patients who met the inclusion criteria for this study were aged 20–98 years, and ultimately, all were adults. We have added this information to the “Study Population” section, as follows:

Our registry did not impose an age limit on patient registration; patients aged 3–101 years were enrolled. However, only patients aged 20–98 years met the inclusion criteria. Consequently, all the eligible patients were adults. (page 7, lines 109-112)

***Comment #4:***

*Line 76 – 78: all the exclusion criteria.*

*Do all samples have complete data that are required for the study? If yes, then ignore my comment. If no, please add it as an exclusion criteria.*

**Reply:**

Not all patients had complete data. Missing values were noted for several variables in Table 1. We have now added this information to both the revised Table 1 and the “Statistical Analysis” section. For analyses that included variables with missing data, multiple imputations were performed using chained equations (MICE; m = 5). These revisions have been added to the “Statistical Analysis” section, as follows:

The proportion of missing data was very low (<5%) across all assessed variables: BMI (1.4%), hemoglobin level (0.4%), platelet count (0.5%), D-dimer level (1.0%), and CRP level (0.7%). To address these missing values, multiple imputations were performed using chained equations (MICE) to generate five imputed datasets. (page 10, lines 159-162)

***Comment #5:***

*Please specify in methodology that the study data was extracted from the TULIPE registry and divided into different sections: Demographic, clinical, investigations, treatment, outcomes… This corresponds to the results reported latter.*

**Reply:**

We have revised the Methods section to clarify that the data were extracted from the TULIPE registry and categorized into predefined domains, including demographics, clinical characteristics, laboratory investigations, treatment details, and clinical outcomes, to ensure consistency with the Results section. This revision has been added to the “Study Design” section, as follows.

To facilitate a structured analysis, data extracted from the TULIPE registry were systematically categorized into predefined domains, including demographics, clinical characteristics, laboratory investigations, treatment information (e.g., anticoagulant type and duration), and clinical outcomes (e.g., VTE recurrence, major bleeding, and overall survival). Demographic data included age, sex, and body mass index (BMI). The clinical characteristics included comorbidities (e.g., hypertension and diabetes), type and location of DVT (proximal or distal), presence of pulmonary embolism (PE), cancer type, and metastatic status. Laboratory findings included hemoglobin, platelet count, D-dimer, and C-reactive protein (CRP) levels, which were selected based on prior evidence of their relevance to VTE recurrence and bleeding risk [10-11]. Treatment-related variables included the type of anticoagulant used (e.g., DOACs or warfarin), duration of therapy, and reasons for discontinuation. (pages 6, lines 82-92)

***Comment #6.***

*The variables for the study were not stated in the methodology. Please spell it out.*

*Example: Patient demographic, clinical, laboratory, and treatment data was collected from the registry.*

*The demographic data include age, gender, BMI…The clinical data include comorbidity, types of DVT, presence of PE… For investigations, it will be better to highlight parameters associated with VTE are selected (this explains why CRP included).*

**Reply:**

As noted in our response to Comment #5, we have revised the Methods section to explicitly list the variables used in this study, grouped under predefined domains, such as demographic, clinical, laboratory, and treatment data. We also included the rationale for selecting laboratory variables, such as CRP level, which has been reported to be associated with VTE recurrence and bleeding risk.

This revision has been added to the “Study Design” section, as follows:

To facilitate a structured analysis, data extracted from the TULIPE registry were systematically categorized into predefined domains, including demographics, clinical characteristics, laboratory investigations, treatment information (e.g., anticoagulant type and duration), and clinical outcomes (e.g., VTE recurrence, major bleeding, and overall survival). Demographic data included age, sex, and body mass index (BMI). The clinical characteristics included comorbidities (e.g., hypertension and diabetes), type and location of DVT (proximal or distal), presence of pulmonary embolism (PE), cancer type, and metastatic status. Laboratory findings included hemoglobin, platelet count, D-dimer, and C-reactive protein (CRP) levels, which were selected based on prior evidence of their relevance to VTE recurrence and bleeding risk [10-11]. Treatment-related variables included the type of anticoagulant used (e.g., DOACs or warfarin), duration of therapy, and reasons for discontinuation. (pages 6, lines 82-92)

***Comment #7:***

*Line 80 – 83: The cancer group was defined as patients with a cancer diagnosis within 6 months prior to the ultrasound; recurrent, regionally advanced, or metastatic cancer; cancer treatment within the previous 6 months; or hematologic cancer not in complete remission (as previously described) [7]*

*The above definition refers to ‘active cancer’ by Raskob et al. However, they also include cancer within 2 years ‘Patients had to have cancer other than basal-cell or squamous-cell skin cancer that was active or had been diagnosed within the previous 2 years and was objectively confirmed’. Therefore, the cancer group in this study is better renamed as active cancer group.*

**Reply:**

We agree that the definition of the cancer group in our study aligns more closely with the criteria for *active cancer*, as described by Raskob et al. We have explicitly specified in the revised manuscript that patients with active cancer constitute the cancer group, whereas those without active cancer constitute the non-cancer group, as follows:

The final study cohort comprised 2,281 patients who were divided into two groups based on the presence or absence of cancer at the time of VTE diagnosis: 1,152 patients with active cancer (cancer group) and 1,129 patients without active cancer (non-cancer group) (Fig 1). (page 7, lines 106-109)

***Comment #8:***

*Line 90 – 91: VTE was defined as new-onset lower extremity venous thrombosis, pulmonary embolism (PE), or Trousseau syndrome, confirmed by imaging.*

*Please cite the articles to support this definition.*

**Reply:**

We have clarified the definition of recurrent VTE, cited an appropriate reference in the revised manuscript, and updated the text as follows:

The primary outcomes were recurrent VTE, major bleeding, and overall survival. Recurrent VTE was defined as new-onset lower-extremity venous thrombosis, PE, or Trousseau syndrome confirmed by imaging (e.g., venous ultrasound or contrast-enhanced computed tomography [CT]), regardless of symptoms, based on previously published criteria [7]. (page 8, line 121-124)

***Comment #9:***

*Overall survival was not defined. Let's say patients die at home or transfer care to another centre and die, does this data capture in your study?*

**Reply:**

As pointed out, the definition of overall survival has not been clearly stated. Overall survival was defined as the number of days from DVT diagnosis to death due to any cause. For patients who were transferred to other hospitals or received home-based care, we attempted to collect outcome data whenever possible by obtaining referral letters or discharge summaries from the treatment institutions. We have now added this definition in the “Clinical Outcomes” section, as follows:

Overall survival (OS) was defined as the number of days from the DVT diagnosis to death from any cause. For patients who were transferred to other hospitals or received home-based care, we attempted to collect outcome data by obtaining referral letters or discharge summaries from the treatment institutions. (page 8, lines 130-133)

Additionally, while we attempted to obtain outcome data by collecting referral letters and discharge summaries from treating institutions whenever possible, we did not conduct follow-ups via telephone or postal contact with patients whose outcome data remained unavailable. This has now been acknowledged as the “limitation” section, as follows:

Fifth, although we obtained referral letters and discharge summaries from patients who experienced major clinical events outside our institution, we did not conduct telephone- or letter-based follow-ups. This may have resulted in the incomplete ascertainment of clinical outcomes. (page 31, lines 514-517)

***Comment #10:***

*Table 3: Anticoagulation therapy beyond the acute phase.*

*Please define the acute phase in methodology.*

**Reply:**

As suggested, we have clearly defined the acute phase in the Methods section. Specifically, anticoagulation therapy beyond the acute phase was defined as continuation beyond 10 days after VTE diagnosis, in accordance with previous literature [Sakamoto J, et al. *Circ J.*2019. PMID: 31548438]. This definition has been added to the “Study Design” section, as follows:

Anticoagulation therapy beyond the acute phase was defined as continuation beyond 10 days after VTE diagnosis, in accordance with the literature [6]. (page 6, lines 93-94)

***Comment #11:***

*Results:
It will be more organized to divide the results into 2 main sections:
1. Baseline: Demographic, clinical, and treatments.
2. Outcomes: VTE recurrence, Bleed, Mortality
So overall results flow is better.*

**Reply:**
As suggested, we have revised the Results section to improve clarity by organizing the content into two main sections.

***Comment #12:***

*Line 118 – 126: Please specify the value (%) followed by p for the variables with*

*significant differences. It was not stated for this part but mentioned for the remaining*

*parts of the result. Please standardise it.*

**Reply:**

We have revised the “Baseline characteristics” section to specify the actual values and corresponding p-values for the variables with significant differences between groups.

The patient characteristics are summarized in Table 1. Several significant differences were observed between the cancer and non-cancer groups. Patients in the cancer group were younger (71 years vs. 72 years, P = 0.018) and had a lower BMI (22.0 kg/m^2^ vs. 22.8 kg/m^2^, P < 0.001). (page 10, lines 167-169)

The cancer group had lower hemoglobin levels (10.7 g/dL vs. 11.1 g/dL, P < 0.001), and higher CRP levels (1.0 mg/dL vs. 0.8 mg/dL, P = 0.002) compared with the non-cancer group. The prevalence of comorbidities, including hypertension (45.7% vs. 55.4%, P < 0.001), dyslipidemia (25.7% vs. 33.1%, P < 0.001), diabetes mellitus (19.0% vs. 25.6%, P < 0.001), and history of VTE (4.1% vs. 8.0%, P < 0.001), was lower in the cancer group. The incidence of PE was higher in the cancer group than in the non-cancer group (10.9% vs. 6.0%, P < 0.001). However, PE severity was lower in the cancer group, as indicated by reduced rates of cardiac arrest or collapse (8.7% vs. 35.3%, P < 0.001), shock, and hypoxemia (19.0% vs. 32.4%, P = 0.038). (pages 10-11, lines 171-178)

***Comment #13:***

*Line 164 – 165, and Line 202 – 204: The adjusted ratio for VTE recurrence and major bleed was based on age, gender, and BMI. By right, the adjusted ratio should be based on covariates with p < 0.05 and deemed significant in other studies. Therefore, in my opinion, it should be: Age, BMI (continuous variables, not BMI as the number is too small), comorbid as a whole, baseline PE, HB level, anticoagulant beyond acute phase, and discontinuation of anticoagulant (even though p not significant, I believe must be related to recurrence of VTE and major bleed in other studies)
Please do the same correction for OS, it was not mentioned in the result.*

**Reply:**

As pointed out, multivariable analyses should consider not only variables with statistically significant p-values but also clinically important covariates supported by previous studies. Therefore, we revised the multivariate models for VTE recurrence, major bleeding, and overall survival (OS) to include the following covariates: age and BMI (as continuous variables), sex, comorbidities as a whole, baseline PE, hemoglobin, anticoagulation beyond the acute phase, and discontinuation of anticoagulation (excluded from the model for major bleeding). Consequently, the subdistribution hazard ratios (sHRs) for VTE recurrence and major bleeding were slightly modified.

- VTE recurrence: from sHR = 2.00 (95% CI: 1.47–2.72) → 2.00 (95% CI: 1.46–2.74)
- Major bleeding: from sHR = 2.85 (95% CI: 2.04–3.96) → 2.69 (95% CI: 1.90–3.81)

We have added this information to the “Statistical Analysis,” “VTE recurrence” and “Major bleeding” sections as follows:

Given the potential impact of mortality as a competing risk, the Fine–Gray subdistribution hazard model was applied to estimate the subdistribution hazard ratios (sHRs) for VTE recurrence and major bleeding. In these models, death from other causes was treated as a competing event, whereas in the Cox model, it was treated as a censored event for comparison. Multivariate models were adjusted for age, sex, BMI, comorbidities as a whole, baseline PE, hemoglobin level, anticoagulation beyond the acute phase, and discontinuation of anticoagulant therapy. Notably, discontinuation was excluded from the adjustment in the major bleeding model because bleeding events occurring after discontinuation were not systematically captured in our registry. Subgroup analyses for VTE recurrence and major bleeding were conducted using Fine–Gray subdistribution hazard models, adjusting for the same covariates in the primary analyses. To evaluate the impact of VTE recurrence and major bleeding on OS, additional subgroup analyses were performed using Cox proportional hazards models, incorporating VTE recurrence or major bleeding as covariates along with the previously described variables. (page 9, lines 144-157)

Considering that mortality may occur before VTE recurrence, mortality was accounted for using the Fine–Gray subdistribution hazard model. After adjusting for confounders, cancer remained an independent predictor of VTE recurrence (adjusted sHR: 2.00; 95% CI: 1.46–2.74; P < 0.001) (Table 5). (page 16, lines 242-245)

Even after adjusting for confounders and considering the competing risk of all-cause death, cancer remained an independent predictor of major bleeding (adjusted sHR: 2.69; 95% CI: 1.90–3.81; P < 0.001), as determined by a Fine–Gray competing risk regression model (Table 7). (page 19, lines 297-300)

***Comment #14:***

*Discussion:
Line 261 – 264: Chatani et al.'s study included only patients with acute symptomatic VTE, whereas our study enrolled patients diagnosed with DVT via ultrasonography, including asymptomatic cases and those with an unknown onset time. Consequently, our cohort had fewer patients with PE or proximal DVT compared to Chatani et al.’s study.
By right, Chatani et al cancer + acute vs acute compared to current study cancer + scan vs scan (those non-cancer groups unlikely have a routine scan, they scan most likely something going on). The outcome should be worse in Chatani. I would rather argue that Chatani et al include active cancer + cancer 6-24 months vs the current study that includes active cancer, leading to dilution of differences in the former. Please read Chatani et al again and see if justifiable. If so, please write the discussion again*

**Reply:**

In the Discussion section, we clarified that our study included patients diagnosed via ultrasonography, including asymptomatic cases, whereas Chatani et al. [Chatani et al., *Thromb Res* 2024. PMID: 38190788] included only patients with acute symptomatic VTE. We also addressed the differences in the definition of cancer and non-cancer groups between the two studies, as their non-cancer group likely included patients with a history of cancer, whereas our study did not. The revised text now reads as follows:
Chatani et al.'s study included only patients with acute symptomatic VTE, whereas our study enrolled patients diagnosed with DVT using ultrasonography, including asymptomatic patients [7]. Consequently, our cohort included fewer cases of PE and proximal DVT than other studies. Moreover, ultrasound in patients without cancer was likely performed based on symptoms and not routine screening, possibly leading to more clinically relevant VTE. Furthermore, in the study by Chatani et al. [7], patients were classified into cancer and non-cancer groups solely based on the presence of active cancer at the time of VTE diagnosis. Therefore, the non-cancer group likely included patients without any history of malignancy and those with a prior history of non-active cancer. In contrast, our non-cancer group was defined strictly as patients without a known history of cancer. This difference in group definitions may have influenced the baseline characteristics and clinical outcomes, and should be considered when comparing the two studies. (page 24, lines 379-389)

***Comment #15:***

*Line 292 – 294: In this study, the discontinuation rate of anticoagulation therapy at one year was high, although no significant difference was observed between the cancer and non-cancer groups (52% vs. 53%,* P *= 0.444).
I would rather argue that discontinuation for non-cancer is falsely high and leads to no different in result. In the non-cancer group, some patients may have discontinuation at 3 to 6 months due to standard practice, while cancer is often life-long. If the number of patients in non-cancer only takes into those who discontinued prematurely before the appropriate period, the exact number may be smaller.*

**Reply:**

We agree that interpretation of the anticoagulant discontinuation rate in the non-cancer group requires careful consideration. As you mentioned, a proportion of patients in the non-cancer group may have appropriately discontinued anticoagulation at 3–6 months in accordance with the clinical guidelines, whereas anticoagulation in cancer patients is often prolonged or indefinite.

Therefore, the overall discontinuation rate at one year may have overestimated the proportion of premature discontinuation in the non-cancer group, potentially masking the true differences between the groups. We have revised the Discussion section to acknowledge this limitation and clarify the interpretation of discontinuation rates in light of the differing clinical contexts as follows:

However, these findings should be interpreted with caution. In the non-cancer group, discontinuation at 3–6 months may be appropriate and consistent with the clinical guidelines for provoked VTE. In contrast, CAT often requires extended or indefinite anticoagulation therapy because of ongoing risk factors. Therefore, comparable discontinuation rates may reflect differences in treatment strategies, rather than true similarities in early termination. This limitation should be considered when interpreting these findings. (page 27, lines 435-440)

***Comment #16:***

*Line 315 – 325: The development of anticoagulants with a lower bleeding risk and improved 316 adherence is essential. Factor XI (FXI) has emerged as a promising therapeutic target for CAT…
This is the clinical implication of this study, please put in a stand alone paragraph.*

**Reply:**

We agree that the discussion of Factor XI (FXI) as a therapeutic target for cancer-associated thrombosis represents an important clinical implication of our study.
Accordingly, we have separated this section into standalone paragraphs to enhance its clarity and emphasis. The information is as follows:

Therefore, the development of anticoagulants with a lower bleeding risk and improved adherence is essential. Factor XI (FXI) has emerged as a promising therapeutic target for CAT because it plays a crucial role in clot formation and has a minimal impact on overall coagulation and hemostasis [37-38]. Currently, FXI inhibitors are being investigated in various clinical settings, including total knee arthroplasty, atrial fibrillation, non-cardioembolic stroke, end-stage renal disease, myocardial infarction, coronavirus disease 2019, and cancer-associated VTE [39]. For the treatment of cancer-associated VTE, two ongoing phase III multicenter randomized controlled trials (RCTs) have evaluated abelacimab, a fully human monoclonal antibody: the ASTER trial (comparing abelacimab to apixaban; NCT05171049) and the MAGNOLIA trial (comparing abelacimab to dalteparin; NCT05171075). The results of these trials may help address the unmet needs in the current management of CAT. (page 29, lines 478-488)

***Comment #17:***

*Study strength: Not mentioned. Please highlight.*

**Reply:**

As pointed out, the strengths of our study have not been explicitly stated. We have added a brief paragraph to the same section on the limitations, describing the strengths of this study. This revision has been made as follows:

This study had several notable strengths. First, it leveraged a large real-world cohort from a tertiary academic center in Japan to provide a contemporary data on cancer-associated VTE during the DOAC era. Second, unlike previous studies that primarily focused on symptomatic VTE, our cohort included both symptomatic and asymptomatic patients, particularly those with isolated distal DVT, which is frequently encountered in oncology settings. Third, we used competing risk models (Fine–Gray) to adjust for the high mortality in patients with cancer, improving the accuracy of VTE and bleeding risk estimates. Fourth, we analyzed the reasons for anticoagulant discontinuation in detail and identified bleeding-related discontinuation as a key predictor of VTE recurrence. Finally, cancer-type–specific risks for both VTE recurrence and major bleeding were explored, offering insights into personalized anticoagulation strategies in patients with cancer. (page 30, lines 491-500)

***Comment #18:***

*Conclusion:
Maybe mentioned a newer agent to address these issues is required?*

**Reply:**

As suggested, we have revised the Conclusion section to briefly mention the need for novel anticoagulant agents with improved safety profiles to address the ongoing challenges associated with cancer-associated VTE. We have added this information to the “Conclusion” section as follows:

These findings underscore the need for novel anticoagulant agents with improved safety profiles for CAT management. (pages 31-32, lines 526-527)

***Comment #19:***

*Tables:
Table 1:
A BMI of more than 35 can remove as the number too small.
Comorbidities as overall, the p should be presented.
For diseases that cannot recover, like chronic lung, heart disease… Please remove the history of, as the disease is still present. Maybe for VTE only is suitable.
Presentation: DVT should be put above, and PE below.*

**Reply:**

Thank you for your detailed suggestion regarding Table 1.
In response:

- We excluded the BMI category of >35 kg/m² because of the small number of cases.
- We have added p-values for comorbidities as an overall category.
- For irreversible conditions such as chronic lung disease and heart failure, we removed the term "history of" and presented them as current comorbidities.
- For clarity, we have moved the row for DVT above that of PE under the “Presentation” section.

These revisions have been implemented in “Table 1” (pages 11-12).

***Comment #20:***

*Table 3:
Ventilator support to be removed. It is irrelevant.*

**Reply:**

As suggested, we have removed “ventilator support” from the revised Table 3.

***Comment #21:***

*Table 4:
DVT should be above PE. Why DVT only, PE with or without DVT? It is rather confusing.
Could it be divided into DVT and PE? Or DVT, PE, DVT + PE?*

**Reply:**

We agree that a clear categorization of recurrence events is important. In our study, we adopted the classification of “DVT only” and “PE with or without DVT” as used in a previous large-scale registry study (the COMMAND VTE Registry [Yamashita Y, et al. *Circ J.*2018. PMID: 29576597]). This approach maintains mutual exclusivity, and reflects the clinical relevance of PE as a severe presentation. To avoid confusion, we have clarified in the footnote in Table 4 that these two categories are mutually exclusive. We hope that this format is acceptable based on established precedents.

^a^“DVT only” and “PE with or without DVT” are mutually exclusive categories. (page 17, line 251)

***Comment #22:***

*Tables 5 and 7: Suggest to correct, including relevant covariates as mentioned in the comment above.*

**Reply:**

As suggested, we have revised the models in Tables 5 and 7 to include the relevant covariates mentioned earlier (age, BMI [continuous], sex, comorbidities, baseline PE, hemoglobin level, anticoagulation beyond the acute phase, and discontinuation of anticoagulants [excluded in the bleeding model]). We updated Tables 5 and 7 accordingly and added a list of covariates to the legends of these tables.

***Comment #23:***

*Figures:
Figure 3 and 5: Why these parameters are selected?
TWC and creatinine clearance were not even mentioned in Table 1. Some don’t have between-group significance like gender, or platelet. The cut-off point of these parameters is also not justified.
I would suggest using the parameters selected for the adjusted ratio as mentioned above, such as:
Age, BMI (continuous variables, not BMI as the number is too small), comorbid as a whole, baseline PE, HB level, anticoagulant beyond acute phase, and discontinuation of anticoagulant (even though p not significant, I believe must be related to recurrence of VTE and major bleed in other studies)*

**Reply:**

We have revised the parameters used in the subgroup analyses presented in Figs 3 and 5 and reanalyzed the data accordingly. The related sections in the Results— “VTE recurrence” and “Major bleeding”—have also been updated to reflect the revised analyses. We have revised the manuscript accordingly and added the following statement to the “VTE recurrence” and “Major bleeding” sections as follows:

Subgroup analyses using multivariable Fine–Gray models showed a consistent trend toward a higher risk of VTE recurrence in patients with cancer than in those without cancer (Fig 3). (pages 17-18, lines 264-266)

Subgroup analyses using multivariable Fine–Gray models showed a consistent trend toward a higher risk of major bleeding events in patients with cancer than in patients without cancer (Fig 5). (pages 20-21, lines 316-318)

**Additional Revisions (not requested by editors and reviewers):**

We identified an error in Fig 1, in which part of the patient flow was originally reported as the number of examinations rather than the number of patients. Accordingly, we have revised Fig 1 and the corresponding description in the manuscript. These corrections did not affect the results or conclusions of this study. These revisions have been added in Fig 1 and the “Study population” section, as follows:

A total of 17,188 lower-extremity venous ultrasound examinations were performed in the TULIPE registry, involving 10,303 patients. (page 7, lines 102-103)
